# Shockwave-driven activation of endoplasmic reticulum stress in osteoblasts to enhance bone formation under osteoporotic conditions

**DOI:** 10.1093/rb/rbaf069

**Published:** 2025-06-27

**Authors:** Dun Luo, Qian Chen, Zhuojie Xiao, Cong Feng, Ruitao Hu, Yuyi Wang, Ce Zhu, Xi Yang, Limin Liu, Xiangfeng Li, Xiangdong Zhu, Yueming Song, Xingdong Zhang

**Affiliations:** National Engineering Research Center for Biomaterials, Department of Orthopedic Surgery and Orthopedic Research Institute, West China Hospital, Sichuan University, Chengdu 610065, China; National Engineering Research Center for Biomaterials, Department of Orthopedic Surgery and Orthopedic Research Institute, West China Hospital, Sichuan University, Chengdu 610065, China; Department of Orthopaedics, Affiliated Hospital of North Sichuan Medical College, Nanchong 637199, China; National Engineering Research Center for Biomaterials, Department of Orthopedic Surgery and Orthopedic Research Institute, West China Hospital, Sichuan University, Chengdu 610065, China; National Engineering Research Center for Biomaterials, Department of Orthopedic Surgery and Orthopedic Research Institute, West China Hospital, Sichuan University, Chengdu 610065, China; Pittsburgh Institute, Sichuan University, Chengdu 610065, China; National Engineering Research Center for Biomaterials, Department of Orthopedic Surgery and Orthopedic Research Institute, West China Hospital, Sichuan University, Chengdu 610065, China; National Engineering Research Center for Biomaterials, Department of Orthopedic Surgery and Orthopedic Research Institute, West China Hospital, Sichuan University, Chengdu 610065, China; National Engineering Research Center for Biomaterials, Department of Orthopedic Surgery and Orthopedic Research Institute, West China Hospital, Sichuan University, Chengdu 610065, China; National Engineering Research Center for Biomaterials, Department of Orthopedic Surgery and Orthopedic Research Institute, West China Hospital, Sichuan University, Chengdu 610065, China; National Engineering Research Center for Biomaterials, Department of Orthopedic Surgery and Orthopedic Research Institute, West China Hospital, Sichuan University, Chengdu 610065, China; National Engineering Research Center for Biomaterials, Department of Orthopedic Surgery and Orthopedic Research Institute, West China Hospital, Sichuan University, Chengdu 610065, China; National Engineering Research Center for Biomaterials, Department of Orthopedic Surgery and Orthopedic Research Institute, West China Hospital, Sichuan University, Chengdu 610065, China; National Engineering Research Center for Biomaterials, Department of Orthopedic Surgery and Orthopedic Research Institute, West China Hospital, Sichuan University, Chengdu 610065, China

**Keywords:** extracorporeal shockwave, osteoporosis, endoplasmic reticulum stress, cellular senescence, osteogenesis

## Abstract

Extracorporeal shockwave (ESW) therapy is a noninvasive physical intervention widely applied in orthopedics for the treatment of musculoskeletal disorders such as plantar fasciitis, osteoarthritis, delayed fracture healing and tendinopathies. In recent years, accumulating evidence has suggested that ESW may also have beneficial effects on bone regeneration and local bone mineral density, particularly under osteoporotic conditions. However, the precise biological mechanisms underlying these effects remain incompletely elucidated. In this study, we systematically investigated the effects of different radial extracorporeal shockwave (r-ESW) intensities on osteoblasts derived from osteoporotic bone (OPOB), with a specific focus on osteogenic activity and the involvement of endoplasmic reticulum (ER) stress. Our *in vitro* results demonstrated that moderate-intensity r-ESW (3 bar) significantly enhanced osteoblast proliferation, upregulated the expression of osteogenic markers including Runx2, Col I, OPN and OCN and promoted matrix mineralization. Mechanistically, this was accompanied by mild ER stress and activation of the PERK-eIF2α-ATF4 signaling pathway, which contributed to improved osteogenic differentiation and alleviated cellular senescence. In contrast, high-intensity stimulation (5 bar) induced excessive ER stress, calcium overload and subsequent apoptosis and necrosis, ultimately impairing osteogenesis. Furthermore, in an ovariectomy (OVX)-induced osteoporotic rat model, 3 bar r-ESW treatment effectively increased bone mass, stimulated new bone formation and decreased osteoclast activity and senescence-associated markers in vivo. These findings collectively highlight the potential of moderate-intensity r-ESW as a promising nonpharmacological strategy for osteoporosis management, providing novel insights into the modulation of ER stress as a therapeutic target in OPOB remodeling.

## Graphical Abstract

**Figure:**
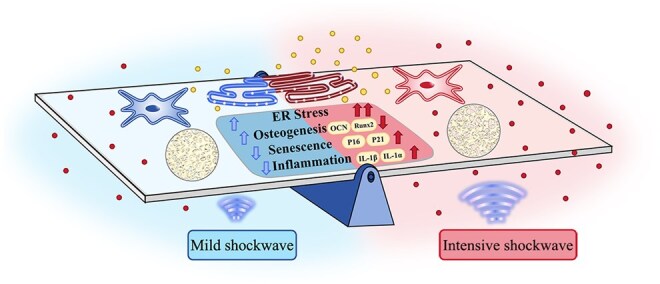

## Introduction

The incidence of osteoporosis continues to rise with an aging global population [1]. This condition substantially elevates the risk of fractures in areas such as the hip, spine and forearm, particularly among the elderly. Osteoporosis represents a significant public health burden, contributing to reduced quality of life and increased healthcare services costs [2]. Research indicates that nearly 50% of women and 20% of men will experience their first osteoporotic fracture after turning 50 [3]. The impact of osteoporotic fractures is profound, being a major cause of disability and mortality in older adults. Studies reveal that among patients sustaining hip fractures due to osteoporosis, 20% succumb to complications within a year, while 50% endure permanent disability [4]. Despite this alarming situation, effective treatment options for osteoporosis remain limited. Although various medications are available for clinical use, a substantial proportion of patients fail to achieve satisfactory outcomes [5]. Moreover, the adverse effects associated with prolonged use of anti-osteoporosis drugs pose significant challenges that require careful attention [6]. Therefore, the treatment of localized osteoporosis requires further exploration.

In recent years, extracorporeal shockwave (ESW) therapy has become increasingly popular in physical therapy and orthopedic medicine, especially for managing disorders such as plantar fasciitis, osteoarthritis and spinal injuries, osteonecrosis of the femoral head and nonunion fractures [7, 8]. ESWs exert therapeutic effects by altering cell signaling through mechanical, cavitation and thermal effects [9]. Recent investigations highlight their ability to improve local bone mineral density in osteoporotic environments, primarily due to their targeted delivery, which ensures precise control over both energy levels and directional focus, optimizing their efficacy in enhancing localized bone density [10–14]. Within bone tissue, studies indicate that the self-renewal capacity and lineage commitment of bone marrow-derived mesenchymal stem cells (BMSCs) and osteoblast-lineage cells are highly responsive to mechanical stimuli [12, 13, 15]. Despite these findings, the exact molecular and cellular mechanisms underlying these effects remain incompletely understood.

A previous study emphasized the pivotal status of endoplasmic reticulum (ER) stress in the pathogenesis of disuse osteoporosis [16]. Recent research has further demonstrated that ESW therapy can induce ER stress, particularly by modulating calcium channels within the ER [17]. Activation of the protein kinase RNA-like endoplasmic reticulum kinase (PERK) pathway by ESW leads to the phosphorylation of both PERK and eukaryotic initiation factor 2α (eIF2α).This cascade of events promotes the upregulation of activating factor 4 (ATF4) expression, which, as a component of the cAMP-responsive element-binding protein (CREB) group of basic leucine zipper transcription factors, regulates several vital processes in osteoblast function [18, 19]. Notably, one of its key transcriptional targets is osteocalcin (OCN), an osteoblast-specific marker indicative of the later stages of osteoblast differentiation [20]. ATF4 is also essential for maintaining the functional integrity of mature osteoblasts, including the synthesis of collagen, the primary extracellular matrix protein in bone tissue [21, 22]. In mice lacking ATF4, a notable decrease or delay in bone mineralization is observed, especially in regions like the frontal and parietal bones, clavicles and long bones [23]. Furthermore, it has been proposed that the PERK-eIF2α-ATF4 signaling pathway is crucial for bone formation and osteoblast differentiation in reaction to ER stress [24]. These results highlight the critical function of ATF4 as a pivotal transcription factor in the final differentiation of osteoblasts and in the process of bone formation.

**Figure 1.**
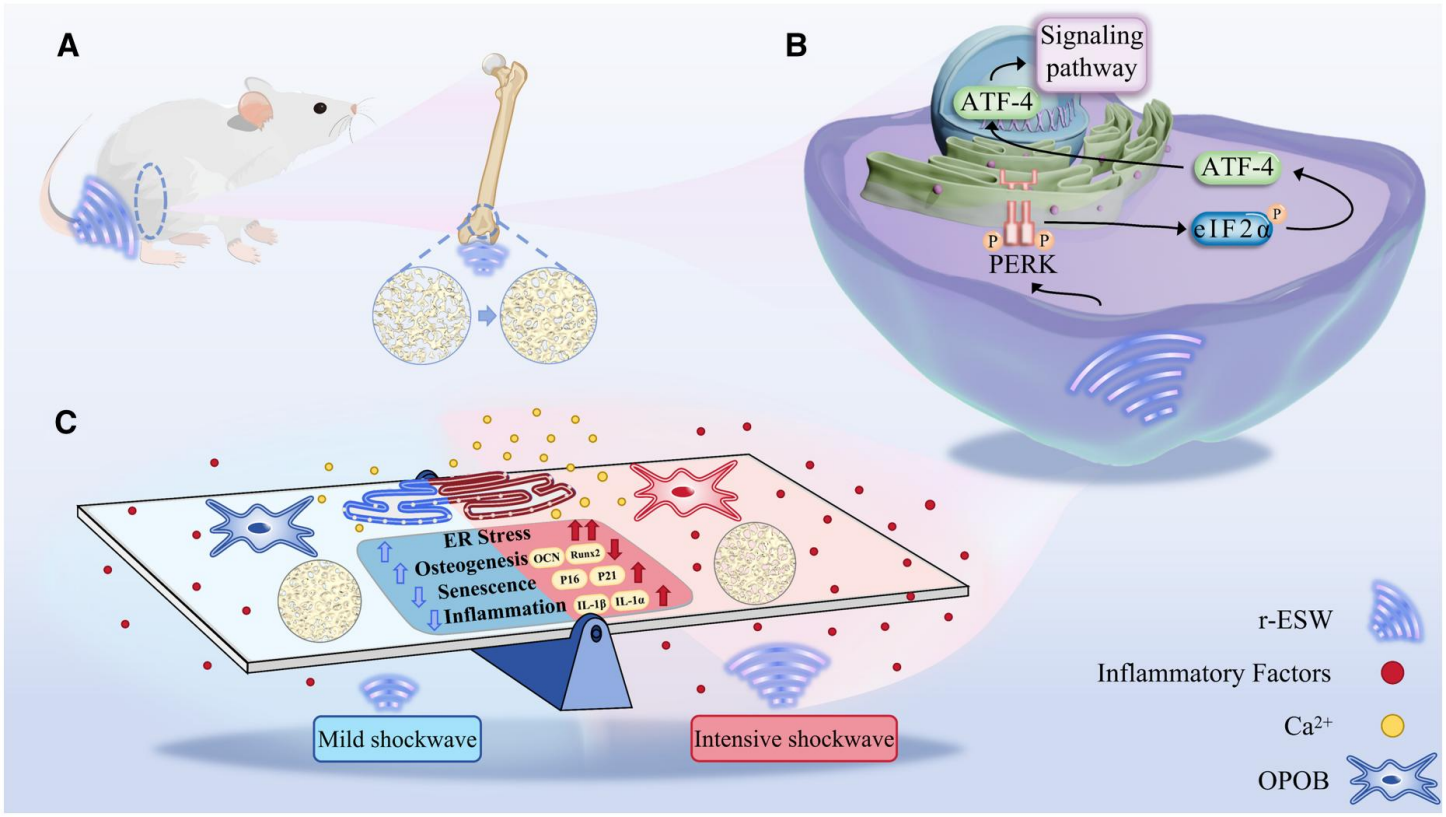
Schematic representation of the effects of r-ESW of different intensities on osteoporotic rats. (**A**) Treatment of osteoporotic rats using r-ESW of different intensities. (**B**) r-ESW altered endoplasmic reticulum-mediated signaling pathways. (**C**) Effects of r-ESW of different intensities on endoplasmic reticulum stress, osteogenesis, senescence and inflammation in osteoporotic rats.

In this study, we sought to explore the impact of ESW treatment on osteoblast differentiation, with a particular focus on the role of endoplasmic reticulum (ER) stress in regulating osteogenesis (Figure 1). Notably, previous studies have mainly modulated ESW energy parameters—including intensity, frequency and pulse number—to indirectly regulate the combined mechanical, cavitation and thermal effects, thereby achieving desired biological outcomes [10, 25]. Based on this, our study also adopted an energy-parameter-based strategy to investigate the influence of r-ESW on ER stress modulation and osteogenesis. Specifically, we sought to examine the impact of ESW treatment on the activation of the PERK-eIF2α-ATF4 signaling pathway, a critical mechanism that governs the expression of osteogenesis-related genes. To evaluate the potential therapeutic effects of ESW on bone regeneration, we utilized both *in vitro* and *in vivo* models, assessing cellular responses and examining key molecular markers critical to osteoblast differentiation and the bone formation process.

## Results and discussion

### Effect of r-ESW gradient intensity on OPOB cell activity

**Figure 2.**
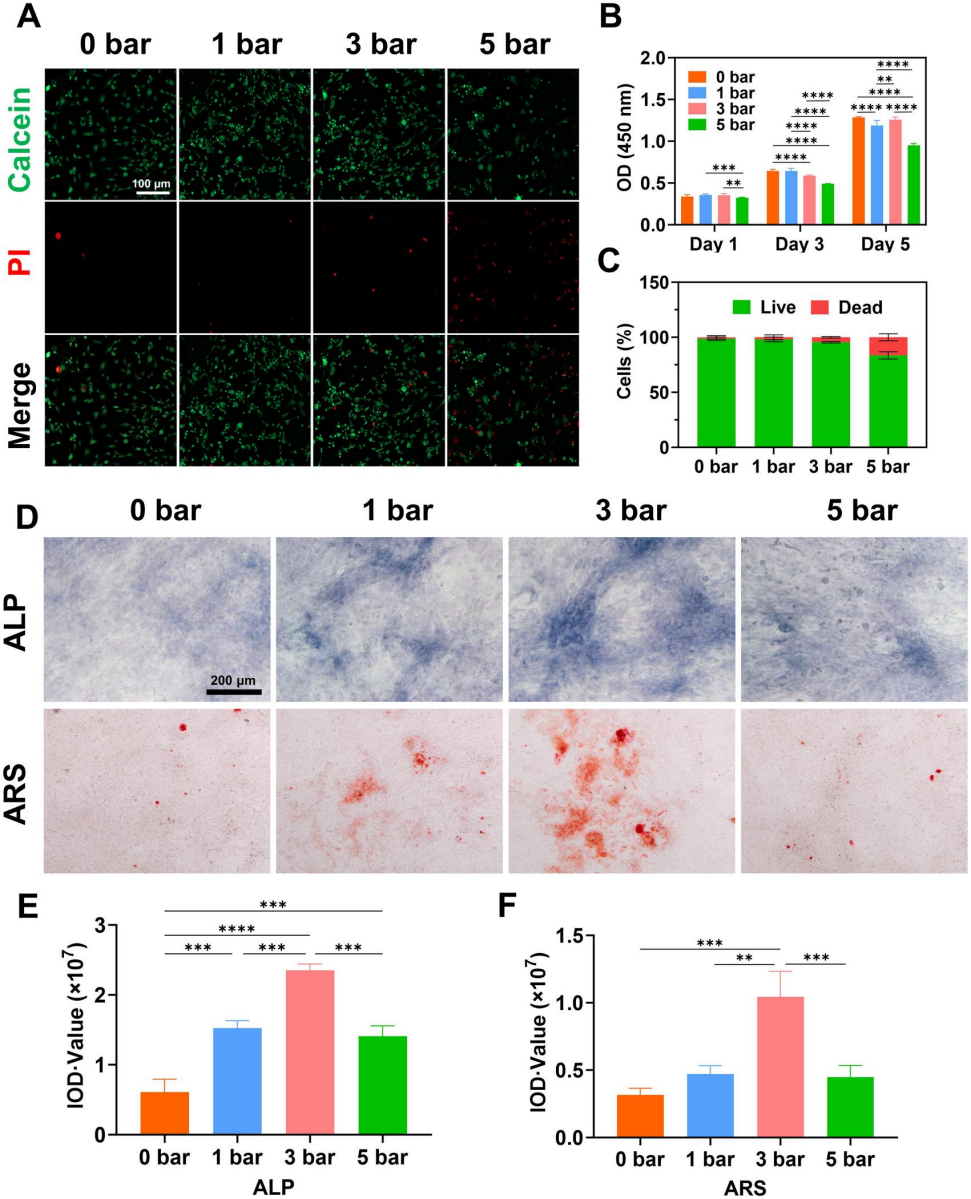
OPOB cell activity and osteogenic effect after treating with different r-ESW intensities. (**A**) Live and dead staining by Calcein-PI (scale bar = 100 μm). (**B**) CCK-8 tests of Day 1, Day 3 and Day 5. (**C**) Quantification of live and dead staining. (**D**) ALP and ARS staining (scale bar = 200 μm). (**E**) Quantification of ALP staining. (**F**) Quantification of ARS staining. The data are presented as the mean ± SD of *n* = 3. The significance of the data was calculated by the one-way analysis of variance (ANOVA). *P ＜ 0.05, **P ＜ 0.01, ***P ＜ 0.001,****P ＜ 0.0001.

Drawing on previous studies, low-intensity shock wave promoted cell proliferation and activity, whereas intensive shock wave exhibited an inhibitory effect [26–28]. We identified distinct shock wave intensity groups: 0 bar, 1 bar, 3 bar and 5 bar groups. In the subsequent *in vitro* and *in vivo* studies, we applied these selected shock wave intensities. Initially, we evaluated how different shockwave intensities influenced osteoporotic bone (OPOB) cell proliferation using the Live/dead staining and quantification (Figure 2A and C). There was significant difference in cell survival rate among groups 0 bar, 1 bar and 3 bar, while 5 bar inhibited cell growth. Results from CCK8 assay was consistent with Live/dead staining (Figure 2B). Low-intensity shock waves (1 bar and 3 bar) did not notably affect the proliferation and viability of OPOB cells. In contrast, intensive r-ESW (5 bar) significantly inhibited cell proliferation and increased the cell death rate. This could be attributed to the mechanical stress induced by intensive shock waves, which exceeded the cells’ tolerance, leading to reduced cell viability and damage [29].

### Effect of r-ESW on OPOB osteogenic ability and selection of optimal intensities

Research has demonstrated that shock waves positively influence cellular bioactivity in osteoporotic conditions [26, 28]. In our research, we investigated the effects of specific shock wave intensities on the osteogenic potential of OPOBs.

Bone alkaline phosphatase, a glycoprotein secreted by osteoblasts, supplies phosphate essential for hydroxyapatite deposition, thus facilitating bone formation. After 14 days of osteogenic induction, the 3 bar group displayed the strongest ALP staining, followed by the 1 bar group, with the 5 bar and control groups showing the weakest staining (Figure 2D and E). Alizarin Red staining also indicated that calcium nodule formation was most prominent in the 3 bar group, greater than in the 1 bar, 5 bar and control groups (Figure 2D and F). The consistency between ALP and Alizarin Red staining results confirmed that shockwave doses of 1 and 3 bar effectively stimulated osteogenic activity in OPOB cells.

**Figure 3.**
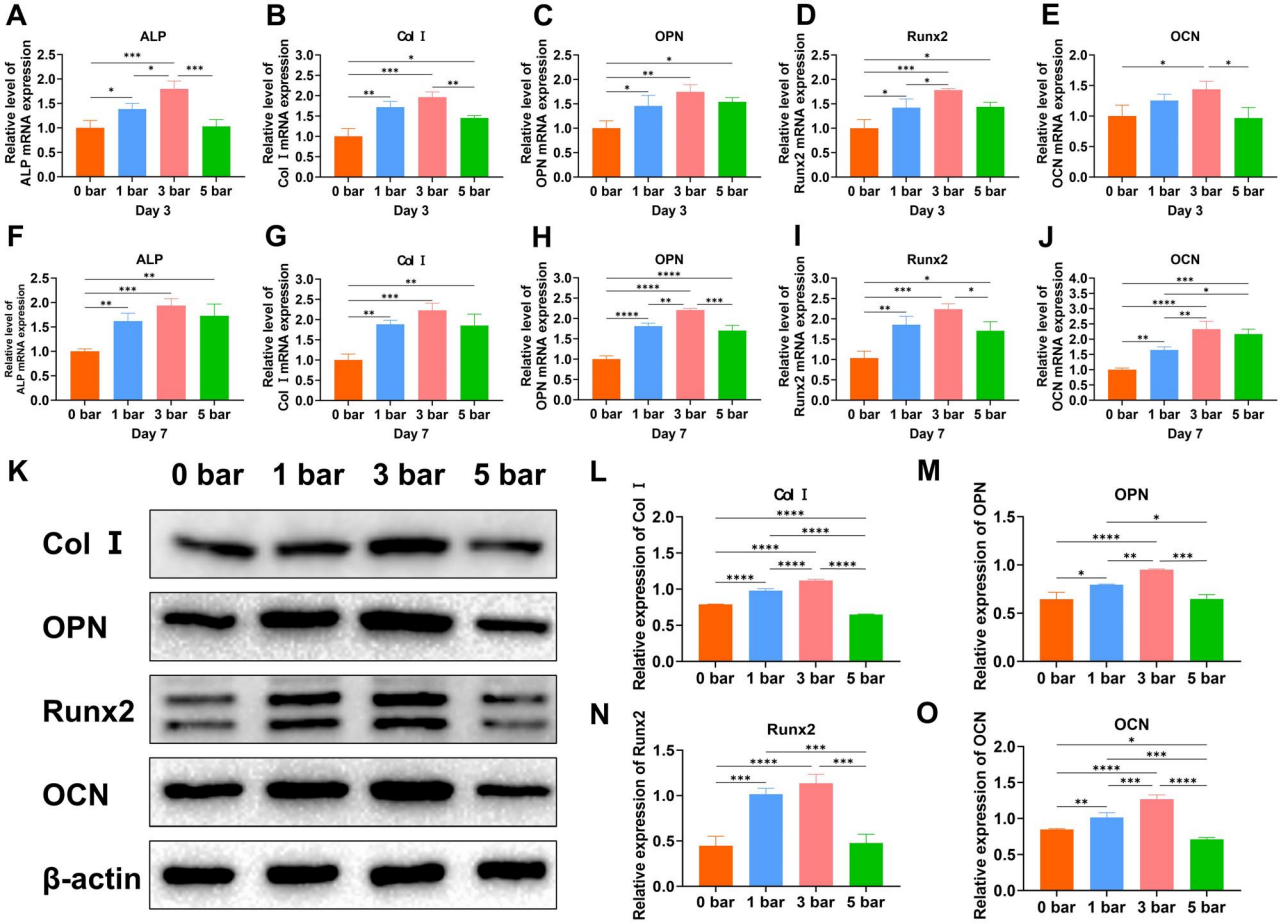
OPOB osteogenic activity after treating with different r-ESW intensities. (**A**–**E**) Relative level of ALP, Col I, OPN, Runx2 and OCN mRNA expression by PCR after 3 days of r-ESW treatment. (**F**–**J**) Relative level of ALP, Col I, OPN, Runx2 and OCN mRNA expression by PCR after 7 days of r-ESW treatment. (**K**) Western blotting of osteogenic markers. (**L**–**O**) Quantification of Col I, OPN, Runx2 and OCN protein expression. The data are presented as the mean ± SD of *n* = 3. The significance of the data was calculated by the one-way analysis of variance (ANOVA). *P ＜ 0.05, **P ＜ 0.01, ***P ＜ 0.001,****P ＜ 0.0001.

In this study, we used RT-qPCR to investigate the effect of varying shockwave intensities on the transcription of osteogenesis-related genes in OPOBs. After 3 days of culturing, transcription levels of osteogenic genes were upregulated across all shockwave-treated groups compared to the control, with the 3 bar group showing the highest upregulation, followed by the 1 bar group (Figure 3A–E). Similar patterns were observed at 7 days, with mRNA transcription in the 3 bar group remaining significantly elevated, suggesting that a 3 bar shockwave dose effectively promoted the transcription of both early and late osteogenic genes (Figure 3F–J).

**Figure 4.**
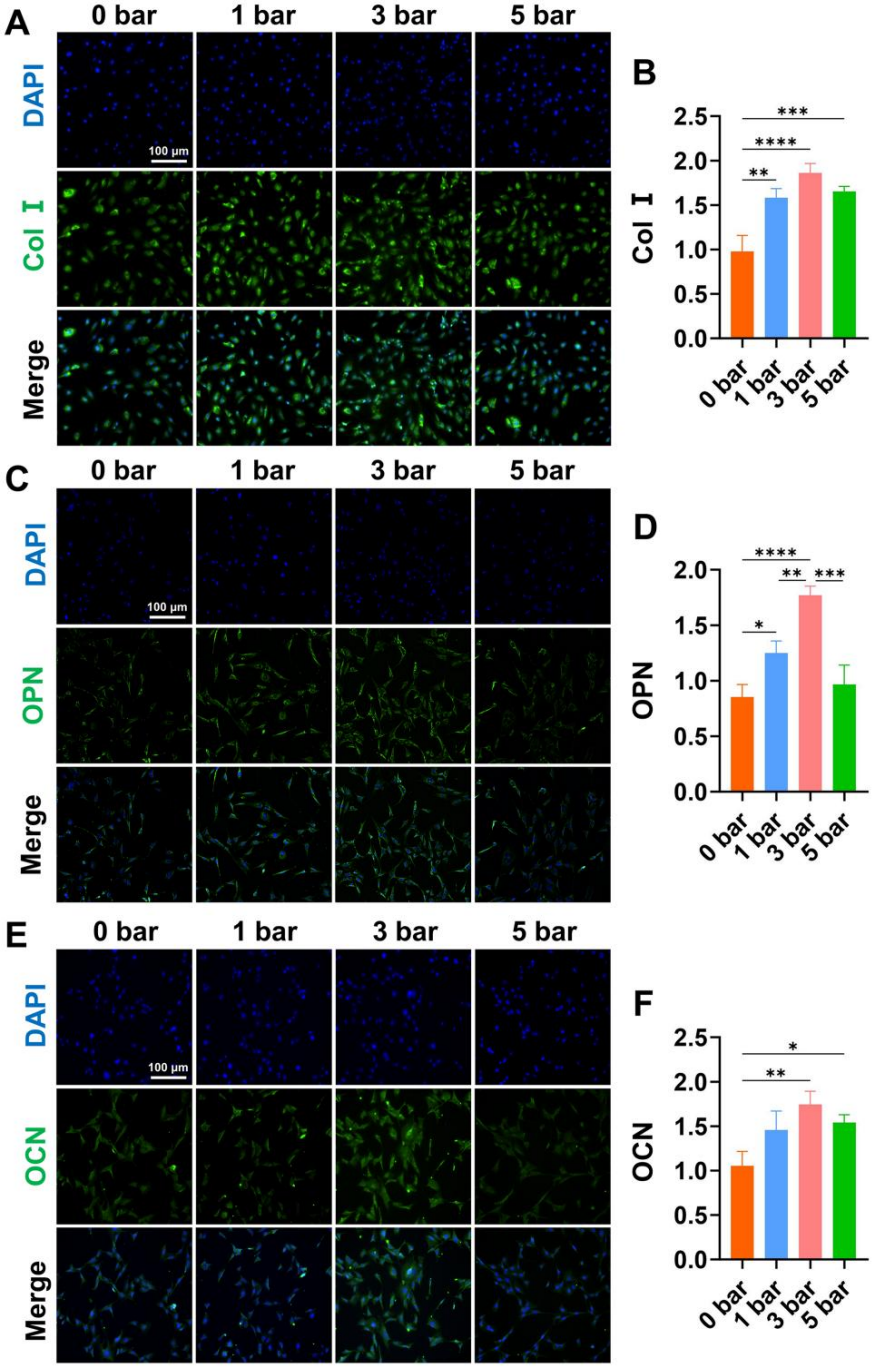
OPOB osteogenic activity after treating with different r-ESW intensities. (**A**) Immunofluorescence of Col I (scale bar = 100 μm). (**B**) Quantification of Col I immunofluorescence. (**C**) Immunofluorescence of OPN (scale bar = 100 μm). (**D**) Quantification of OPN immunofluorescence. (**E**) Immunofluorescence of OCN (scale bar = 100 μm). (**F**) Quantification of OCN immunofluorescence. The data are presented as the mean ± SD of *n* = 3. The significance of the data was calculated by the one-way analysis of variance (ANOVA). *P ＜ 0.05, **P ＜ 0.01, ***P ＜ 0.001,****P ＜ 0.0001.

We also conducted Western blot analysis to quantify osteogenic protein expression. Densitometric analysis of Western blot bands, normalized to β-actin, showed that both 1 bar and 3 bar r-ESW intensities enhanced the expression levels of osteogenic proteins, including Col I, OPN, Runx2 and OCN, compared with 0 bar group. Notably, the 3 bar intensity exerted a more pronounced effect. However, an excessive intensity of 5 bar resulted in lower expression levels of these osteogenic markers compared to the 3 bar group (Figure 3K–O). In immunofluorescence analysis, the 3 bar group exhibited the strongest fluorescence intensity for Col I, OPN and OCN, suggesting that this shock wave intensity not only stimulated osteogenic gene transcription but also increased the expression of osteogenic proteins. These findings were consistent with the results obtained from Western blot analysis, further supporting the conclusion that 3 bar shock waves had a more pronounced effect on osteoblastic maturation and mineralization compared to lower and higher shock wave doses (Figure 4A–F).

Although this study focused on OPOBs, the osteogenic effects of 3-bar ESWT on other cell types (e.g. BMSCs) have been confirmed in prior research [10, 30, 31]. Lv *et al*. reported that 3-bar ESWT enhanced BMSC osteogenic differentiation via mechanical stimulation and cavitation effects [30]; Inoue *et al*. demonstrated accelerated bone repair through BMSC-mediated intramembranous ossification in an osteoporotic defect model [10]; Schleusser *et al*. showed increased VEGF secretion by BMSCs, improving local angiogenesis [31]. Collectively, these findings suggest that the osteogenic benefits of 3-bar ESWT may involve multi-cellular synergistic effects.

Our results indicated that shock waves enhanced osteogenic effects in OPOBs in an intensity-dependent manner under low-intensity pressure. Low-intensity shock waves (1 bar and 3 bar) promoted osteoblastic activity, including the transcription of osteogenic genes and the expression of osteogenic proteins, whereas shock waves exceeding a certain threshold, such as intensive shock waves (5 bar), exhibited an inhibitory effect.

The transition from a promotive to an inhibitory effect with varying shock wave intensities may arise from multiple factors. First, the mechanical stress induced by shock waves could cause ER stress. However, when the mechanical stress is too high, the function of the ER may be disrupted, leading to the accumulation of misfolded proteins and triggers the activation of the ER stress response pathway [32]. When this exceeds the physiological tolerance of osteoblasts, it may result in further damage, including cell membrane rupture and an abnormal increase in intracellular calcium ion concentration, triggering apoptosis or necrosis. Excessive shock wave energy can also trigger oxidative stress in cells, raising the levels of free radicals and reactive oxygen species. This in turn leads to damage of cellular proteins, lipids and DNA, which ultimately results in cellular dysfunction or senescence [33]. These factors may collectively contribute to the inhibitory effect of intensive shock waves on OPOB cells. The mechanism underlying the osteogenic promotion observed at 3 bar intensity will be further analyzed through transcriptomic sequencing.

### r-ESW enhances osteogenic capacity by activating PERK-eIF2α-ATF4 pathway-mediated ER stress and reducing cellular senescence

**Figure 5.**
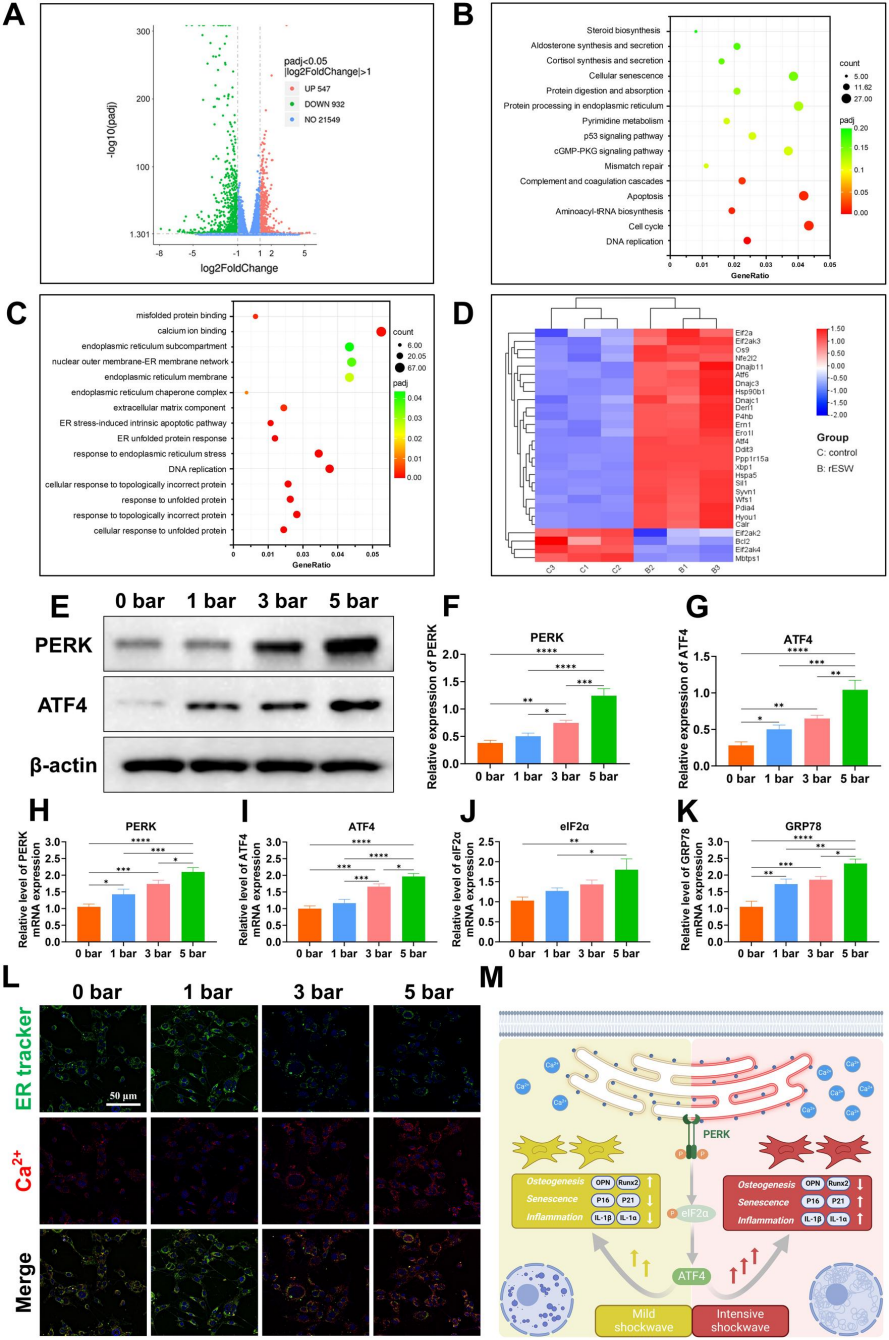
OPOB endoplasmic reticulum stress after treating with different r-ESW intensities. (**A**) Volcano plots revealed down-regulation and up-regulation of genes of 3 bar vs. 0 bar. (**B**) Scatter plot shows the enrichment significance and the number of differentially expressed genes for 15 significant GO terms from the GO enrichment analysis. (**C**) Scatter plot shows the enrichment significance and the number of differentially expressed genes for the significant pathways from the KEGG enrichment analysis. (**D**) Heatmap showed the differentially expressed genes in the protein processing in endoplasmic reticulum pathway from the KEGG enrichment analysis. (**E**) Western blotting of endoplasmic reticulum related markers. (**F** and **G**) Quantification of PERK and ATF4 protein expression. (**H**–**K**) Relative level of PERK, ATF4, eIF2α and GRP78 mRNA expression. (**L**) Endoplasmic reticulum and Ca^2+^ staining (scale bar = 50 μm). (**M**) Schematic diagram of OPOB endoplasmic reticulum stress and osteogenesis according to different r-ESW intensities. The data are presented as the mean ± SD of *n* = 3. The significance of the data was calculated by the one-way analysis of variance (ANOVA). *P ＜ 0.05, **P ＜ 0.01, ***P ＜ 0.001,****P ＜ 0.0001.

The GO enrichment analysis of biological processes identified ten GO terms with significant alterations. These included proteins associated with cellular responses to unfolded or misfolded proteins, as well as those involved in DNA replication, endoplasmic reticulum stress and the unfolded protein response within the endoplasmic reticulum, among others (Figure 5A and B and Supplementary Figure S1). As expected, proteins associated with the endoplasmic reticulum membrane were significantly upregulated in groups subjected to 3 bar pressure compared to the control groups, whereas proteins involved in cell proliferation displayed a downward trend.

Endoplasmic reticulum stress is considered a protective cellular response to various physiological and pathological disturbances that disrupt ER homeostasis [34]. It triggers the unfolded protein response (UPR), activating three major signaling routes: the PERK-eIF2α-ATF4 pathway, the IRE1-XBP1 pathway and the ATF6 signaling pathway [35]. These pathways mitigate the protein-folding load and restore ER balance by inducing the expression of molecular chaperones, such as GRP78 and ER stress sensors, including PERK, IRE1 and ATF6 [36–38].

To gain deeper insights into these processes, KEGG (Kyoto Encyclopedia of Genes and Genomes) pathway enrichment analysis was performed to pinpoint the associated pathways. As shown in Figure 5C and D and Supplementary Figure S2, multiple signaling pathways related to ER stress were found to be enriched. Notably, proteins involved in the ER stress response, including PERK and ATF4, were upregulated during protein processing in the ER. In response to ER stress, the buildup of unfolded or misfolded proteins activates PERK, a key sensor in the unfolded protein response (UPR). Once activated, PERK phosphorylates eIF2α, leading to the suppression of general protein synthesis to alleviate the stress on the ER [39]. Concurrently, phosphorylated eIF2α promotes the selective translation of ATF4, which in turn activates the expression of genes associated with the ER stress response. Our results confirmed that r-ESW treatment upregulated ER stress-related pathways, in line with our expectations.

We also evaluated how varying r-ESW intensities affect the ER stress response mediated by the PERK-eIF2α-ATF4 pathway, focusing on the expression of related genes and proteins. After r-ESW treatment, both protein and mRNA expression levels of PERK and ATF4 were upregulated compared with 0 bar group, normalized to β-actin, with the highest increase observed in the 5 bar group (Figure 5E–I). Furthermore, the mRNA levels of eIF2α and GRP78, important markers of ER stress, were notably elevated in the 5 bar group relative to the other groups (Figure 5J and K). These results suggested that r-ESW treatment, particularly at 5 bar, strongly activated the PERK-eIF2α-ATF4 pathway, indicating a dose-dependent enhancement of ER stress in response to increasing r-ESW intensity.

During the ER stress response, the morphology of the ER can experience swelling, dilation and potentially membrane rupture. Additionally, calcium channels within the ER are activated, leading to the release of large amounts of calcium ions into the cytoplasm. To examine ER morphology and calcium ion distribution, we used ER-Tracker Green and calcium ion staining techniques (Figure 5L). Colocalization staining was employed to evaluate ER stress severity and the depletion of ER calcium stores. The experimental data revealed that, in the 5 bar treatment group, ER fluorescence diminished markedly, with disrupted localization and fragmented structures, along with a substantial calcium influx into the cytoplasm. This elevated calcium release, known to intensify ER stress [40], suggested a more severe ER stress response compared to the 3 bar group. Wang *et al*. demonstrated that the osteogenic and angiogenic effects induced by r-ESW are, in part, mediated by the activation of the Piezo1/Ca^2+^/CaMKII/CREB signaling pathway [28]. This mechanism involves the activation of the Piezo1 channel, which promotes the influx of intracellular calcium ions, thereby initiating downstream signaling events. The influx of calcium subsequently promotes the expression of critical markers related to osteogenesis and angiogenesis, such as Runx2 and OCN [28]. In our study, we observed that the calcium ion efflux in the 3 bar r-ESW group was significantly lower than that in the 5 bar group. This disparity may suggest a potential connection between ER stress, calcium ion regulation and osteogenesis.

ATF4, a downstream effector of PERK, is implicated in regulating various bone-forming factors and osteogenic genes. Saito *et al*. conducted a study showing that ER stress, driven by the PERK-eIF2α-ATF4 pathway, is essential for osteoblast differentiation stimulated by BMP2 [20, 41]. Additionally, activation of the PERK-eIF2α-ATF4 pathway enhances the expression of ATF4 target genes, such as OCN, which is a marker of mature osteoblasts. This finding aligns with previous research showing that shockwave treatment promotes osteogenesis by increasing OCN expression. Additionally, other research has suggested that the activity of ATF4 is mainly controlled by the Wnt/β-catenin signaling pathway [15]. BMP2 is known to upregulate Wnt signaling, thereby further enhancing osteoblast differentiation. Given this, it can be hypothesized that ER stress is closely linked to key force transduction pathways in bone cells, such as the Wnt pathway. However, this relationship warrants further investigation.

Notably, excessive release of calcium ions is known to intensify ER stress and disturbances in intracellular Ca^2+^ balance can induce apoptosis via ER stress pathways [42, 43]. Therefore, we hypothesize that the excessive activation of ER stress at a pressure level of 5 bar may be responsible for the increased necrosis observed in OPOBs.

**Figure 6.**
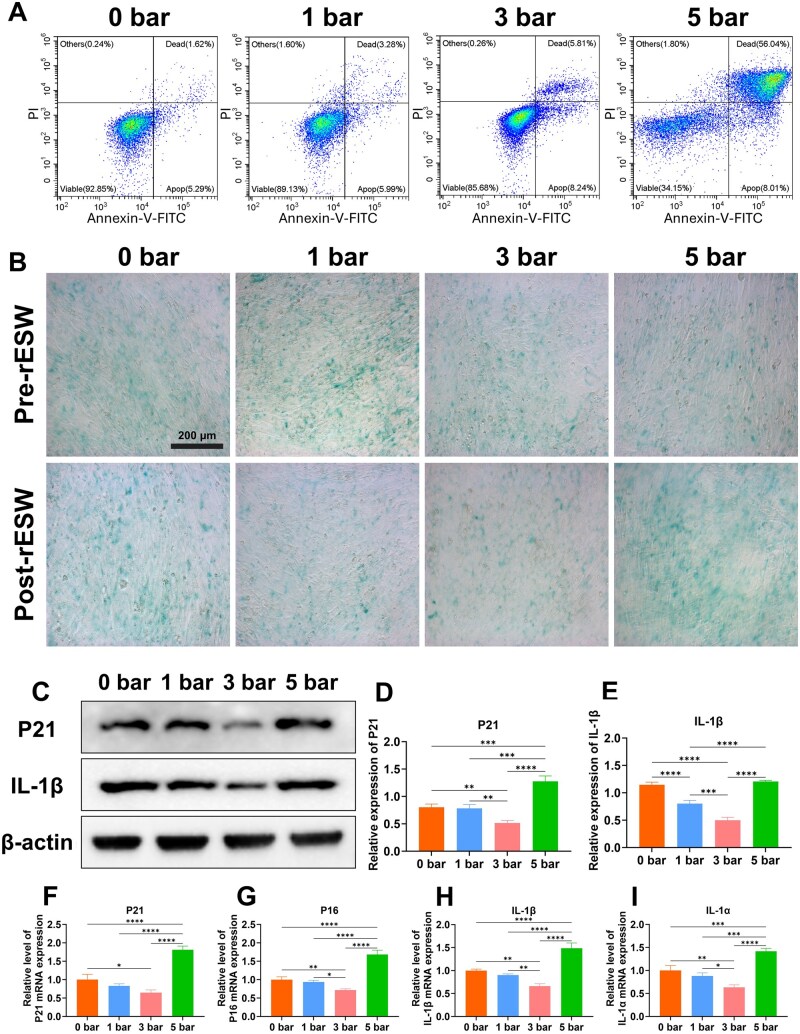
OPOB senescence and inflammation after treating with different r-ESW intensities. (**A**) Apoptosis of OPOB after treating with different r-ESW intensities. (**B**) The senescence of OPOB by β-galactosidase (SA-β-gal) staining (scale bar = 200 μm). (**C**) Western blotting of senescent and inflammatory markers. (**D** and **E**) Quantification of P21 and IL-1β protein expression. (**F**–**I**) Relative level of P21, P16, IL-1β, IL-1α mRNA expression. The data are presented as the mean ± SD of *n* = 3. The significance of the data was calculated by the one-way analysis of variance (ANOVA). *P ＜ 0.05, **P ＜ 0.01, ***P ＜ 0.001,****P ＜ 0.0001.

Consistent with our initial hypothesis, as the Annexin-V-FITC flow cytometry cell apoptosis detection showed, the application of 5 bar r-ESW to OPOBs led to marked cellular necrosis, corroborated by Calcein AM/PI assay results (Figure 6A). It is important to emphasize that cells treated at 1 bar and 3 bar intensities displayed a gradual increase in apoptosis rates. Previous studies have shown that r-ESW can inhibit apoptosis *in vivo* [44]. However, some level of apoptotic response was observed under 3 bar treatment in practical experimental conditions. While a low degree of ER stress can help restore cellular balance [45], prolonged or unresolved stress in the ER ultimately results in apoptosis. Based on these findings, we found that the occurrence of apoptosis may be associated with escalating r-ESW intensity. Studying the effects of lower r-ESW intensity or energy flux density may provide further valuable insights.

Several mechanisms mediate the changes in aging bone that lead to osteoporosis, with the accumulation of senescent cells being one of the main contributors [46]. OPOBs subjected to various r-ESW intensities were analyzed *in vitro* to assess their senescence status. Senescence-associated β-galactosidase (SA-β-gal) staining results revealed that senescent cells, marked by aquamarine blue staining (SA-β-Gal^+^), showed different responses after r-ESW treatment in OPOBs originally isolated from OVX-induced rats. As depicted in Figure 6B, treatment with 3 bar r-ESW reduced the percentage of β-galactosidase-positive senescent cells compared to baseline levels, while the 1 bar intensity had no significant effect on the proportion of stained senescent cells. Conversely, exposure to 5 bar r-ESW increased the percentage of SA β-Gal^+^ cells.

In the 3 bar treatment group, the expression of senescence markers and SASP factors exhibited distinct alterations. Quantitative analysis of Western blot results, normalized to β-actin, demonstrated that 3 bar treatment significantly reduced the expression of P21 and IL-1β, with the protein band intensities notably weaker compared to the 0 bar group and other treatment groups (1 bar and 5 bar) (Figure 6C–E). In contrast, the 5 bar treatment group demonstrated a marked upregulation of P21 and P16 protein expression, with levels significantly higher than those observed in the 0 bar group. These findings were corroborated at the transcriptional level, with qPCR results showing consistent patterns (Figure 6F–H). Specifically, the mRNA expression of P21 and IL-1β in the 3 bar group was significantly lower than in the other treatment groups. Furthermore, the mRNA expression of P16 also decreased in the 3 bar group (Figure 6G). The inflammation-related gene IL-1α was similarly downregulated in the 3 bar group (Figure 6I). These results suggested that moderate mechanical pressure (3 bar) may improve the inflammatory microenvironment by modulating SASP expression. In contrast, the 1 bar group showed minimal changes in these markers, indicating that such a low pressure is insufficient to significantly impact cellular senescence. On the other level, the 5 bar treatment group showed a pronounced upregulation of P21, P16, IL-1β and IL-1α, suggesting that higher pressure may exacerbate cellular senescence and worsen the inflammatory microenvironment.

In summary, the 3 bar treatment significantly suppressed the expression of senescence-related genes (e.g. P21, P16) and SASP factors (e.g. IL-1β, IL-1α). These changes in gene and protein expression may reflect the adaptive response of cells to mechanical stress, particularly by downregulating key genes like P21 and IL-1β, which are involved in cell cycle regulation and inflammatory pathways. This highlights the potential of 3 bar treatment to improve or reverse the senescent microenvironment. In line with previous studies, Mario proposed that ESWT has the potential to counteract cellular senescence and the production of inflammatory cytokines [47]. In contrast, 5 bar treatment aggravated the expression of senescence-related genes, indicating that moderate pressure (3 bar) might be the optimal condition for modulating senescence and inflammatory responses. Collectively, our findings indicate that ER stress plays a vital role in regulating cellular senescence in osteoporotic conditions.

In conclusion, our findings suggest that the intensity of r-ESW has a profound effect on endoplasmic reticulum (ER) stress by activating the PERK-eIF2α-ATF4 pathway, which in turn regulates downstream osteogenic pathways. Specifically, when the r-ESW intensity exceeds a certain threshold, it leads to enhanced calcium ion release and disruption of intracellular Ca^2+^ homeostasis, potentially amplifying ER stress and triggering cellular apoptosis or even necrosis. In contrast, moderate ER stress seems to have a beneficial effect on the senescent microenvironment. A summary of the proposed mechanisms underlying the effects of r-ESW is provided in Figure 5M. Additionally, previous research has highlighted the crucial role of ER stress in regulating osteoclast apoptosis in osteoporotic conditions [45], a hypothesis we plan to validate through further *in vivo* studies.

### Effect of r-ESW on the improvement of osteoporosis *in vivo*

**Figure 7.**
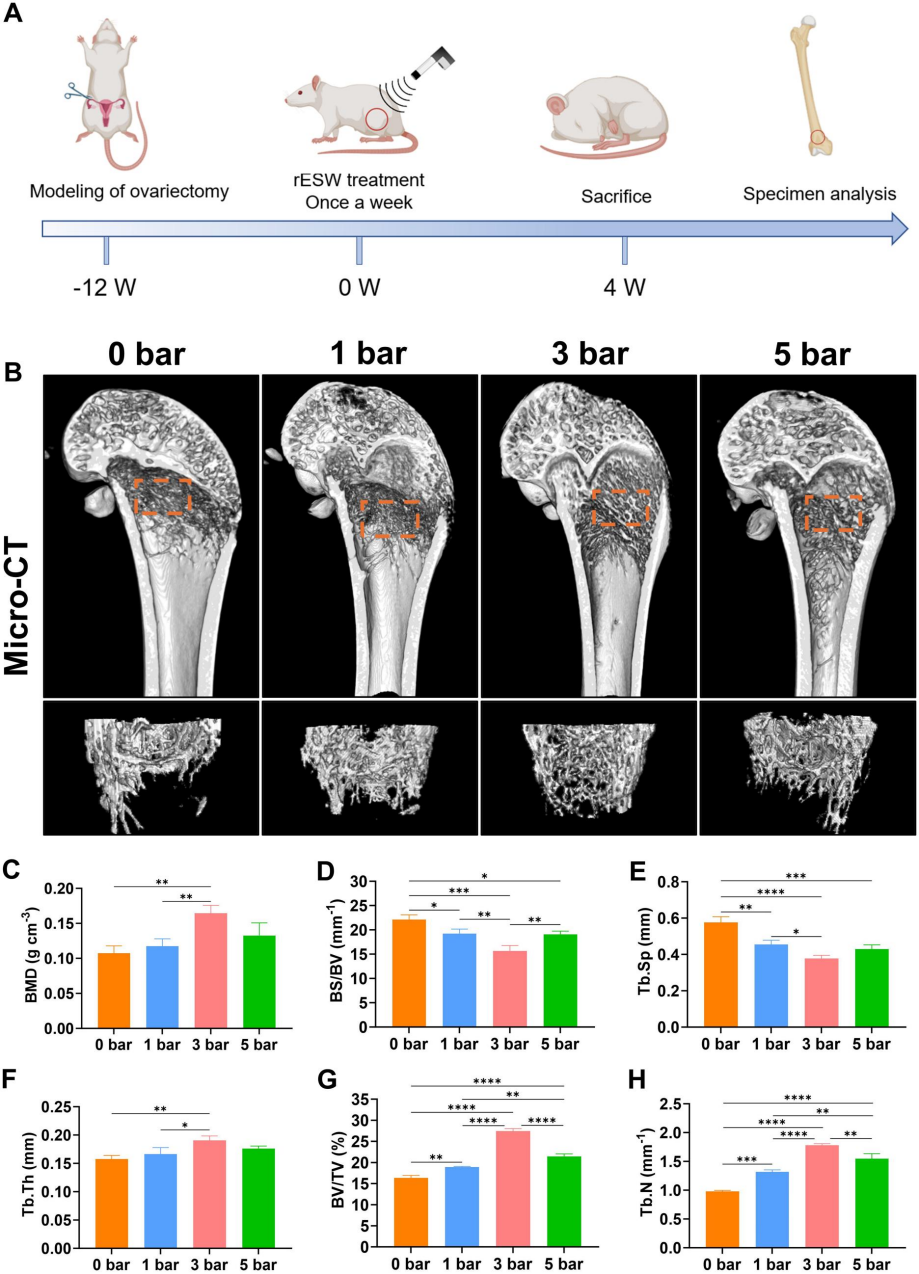
Construction and reparation of osteoporosis. (**A**) Flow diagram of model and treatment of osteoporotic bone. (**B**) The microcomputed tomography of femur after different r-ESW intensities treatment. (**C**) Quantitative analysis of bone mineral density (BMD). (**D**) Quantitative analysis of bone surface/bone volume (BS/BV). (**E**) Quantitative analysis of trabecular separation (Tb.Sp). (**F**) Quantitative analysis of trabecular thickness (Tb.Th). (**G**) Quantitative analysis of bone volume/total volume (BV/TV). (**H**) Quantitative analysis of trabecular number (Tb.N). The data are presented as the mean ± SD of *n* = 3. The significance of the data was calculated by the one-way analysis of variance (ANOVA). *P ＜ 0.05, **P ＜ 0.01, ***P ＜ 0.001,****P ＜ 0.0001.

It is important to note that the use of precisely controlled energy parameters—including intensity, frequency and pulse number—has been widely adopted in previous studies to regulate ESW-induced biological effects [10, 25]. Based on this, we first screened intensities of 0, 1, 3 and 5 bar *in vitro* and identified 3 bar as the optimal parameter. To ensure consistency and facilitate clinical translation, the same intensity gradient was subsequently applied *in vivo*, aligning with parameter settings validated in prior studies [10, 25]. To investigate the impact of r-ESW on osteoporosis, we utilized an ovariectomy (OVX)-induced osteoporotic rat model, which underwent r-ESW treatment for a duration of four weeks (Figure 7A). Our results indicated that r-ESW alleviates senile osteoporosis, at least partially, by inducing a moderate ER stress response via the PERK-eIF2α-ATF4 pathway, which is crucial for osteoblast differentiation *in vivo*. Micro-computed tomography (micro-CT) analysis demonstrated a notable increase in trabecular bone mass in OVX rats treated with 3 bar r-ESW (Figure 7B). We utilized micro-CT to examine the trabecular microstructure of the distal femoral epiphysis in OVX rats, evaluating the effects of r-ESW treatment (Figure 7C–H). The results showed that the 3 bar r-ESW group exhibited substantial improvements across several trabecular parameters. Specifically, the 3 bar group exhibited a significant increase in bone mineral density (BMD) (0.163 ± 0.53 g·cm^−^³) when compared to both the control and 1 bar groups, along with enhanced trabecular thickness (Tb.Th), trabecular number (Tb.N) and the bone volume to tissue volume ratio (BV/TV), with a notable 27.4% improvement in BV/TV (*P* < 0.05). Additionally, trabecular separation (Tb.Sp) and the bone surface to bone volume ratio (BS/BV) were significantly reduced. These findings suggest that 3 bar r-ESW improves trabecular structure, density and quality, thereby enhancing the mechanical properties of bone tissue and partially reversing osteoporotic pathology. Although the 5 bar group showed some improvements in Tb.Sp and Tb.N, the overall effect was less pronounced than that of the 3 bar group, indicating that excessive r-ESW intensity may limit or even inhibit improvements in trabecular quality. Overall, these experimental results supported the hypothesis that r-ESW treatment induces mild ER stress through the PERK-eIF2α-ATF4 pathway, which promotes osteogenesis. However, excessive or prolonged ER stress can lead to cellular damage and apoptosis, ultimately diminishing the osteogenic effect.

**Figure 8.**
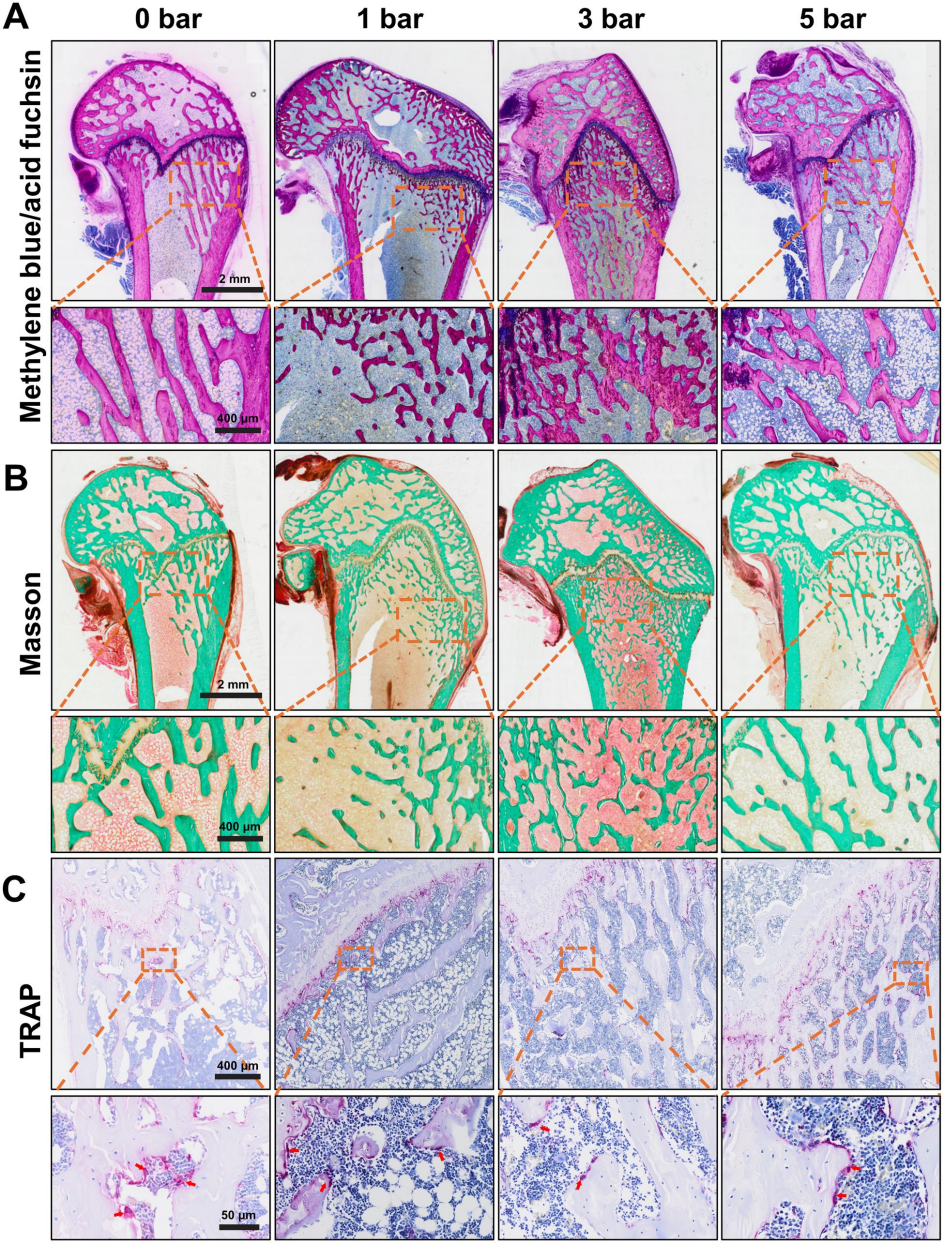
Histological evaluation of osteoporosis reparation *in vivo* after r-ESW treatment. (**A**) Representative images of histological staining (Methylene Blue/Acid Fuchsin staining) of osteoporotic femurs after treating with different r-ESW intensities. (**B**) Representative images of Masson trichrome histochemical staining of osteoporotic femurs after treating with different r-ESW intensities. (**C**) Representative images of tartrate-resistant acid phosphatase (TRAP) staining of osteoporotic femurs after treating with different r-ESW intensities.

The results were further corroborated by Methylene Blue/Acid Fuchsin staining, which revealed a marked increase in the red staining, indicating enhanced mineralization of new bone tissue in the 3 bar group. Additionally, the extent of blue staining, representing newly deposited bone matrix, was also more extensive, suggesting a significant deposition of new bone material. These findings indicated that the 3 bar intensity treatment notably promoted both new bone formation and mineralization under osteoporotic conditions (Figure 8A). Moreover, as illustrated in Figure 8B, Masson staining at four weeks demonstrated increased collagen matrix deposition, with the 3 bar r-ESW treatment in OVX-induced rats significantly enhancing new bone formation compared to the other groups. The results demonstrated a fold increase in new bone formation in the 3 bar group compared to the 1 bar, 5 bar and control groups. This study determined the optimal parameters for our experimental system (3 bar, 5 Hz, 500 impulses). Wang *et al*. previously demonstrated that low-energy r-ESW minimizes cellular damage [28], which aligns with our findings. In contrast, Chen *et al*. found that a high energy flux density (0.50 mJ·mm^−2^) resulted in the opposite effect, worsening cellular damage [26]. These observations were consistent with our results. Furthermore, although the precise mechanisms by which cells perceive mechanical stimuli remain unclear, further investigation is needed to fully elucidate the underlying signaling pathways involved.

Notably, the number of TRAP-positive cells decreased in the 3 bar r-ESW treated group, suggesting an inhibitory effect of r-ESW on osteoclast activity (Figure 8C). Osteoporosis, as previously outlined, arises from excessive bone resorption and disrupted bone remodeling, often linked to heightened osteoclastogenesis and osteoclast activity [45, 48, 49]. Our *in vivo* results indicated that 3 bar r-ESW successfully stimulated new bone formation and mitigated OVX-induced osteoporosis, likely due to a reduction in osteoclast numbers. However, the underlying mechanisms contributing to these effects remain under investigation. Consequently, we propose that r-ESW targeting the elimination of osteoclasts could be an effective strategy to boost bone formation capacity in aged bone tissue.

**Figure 9.**
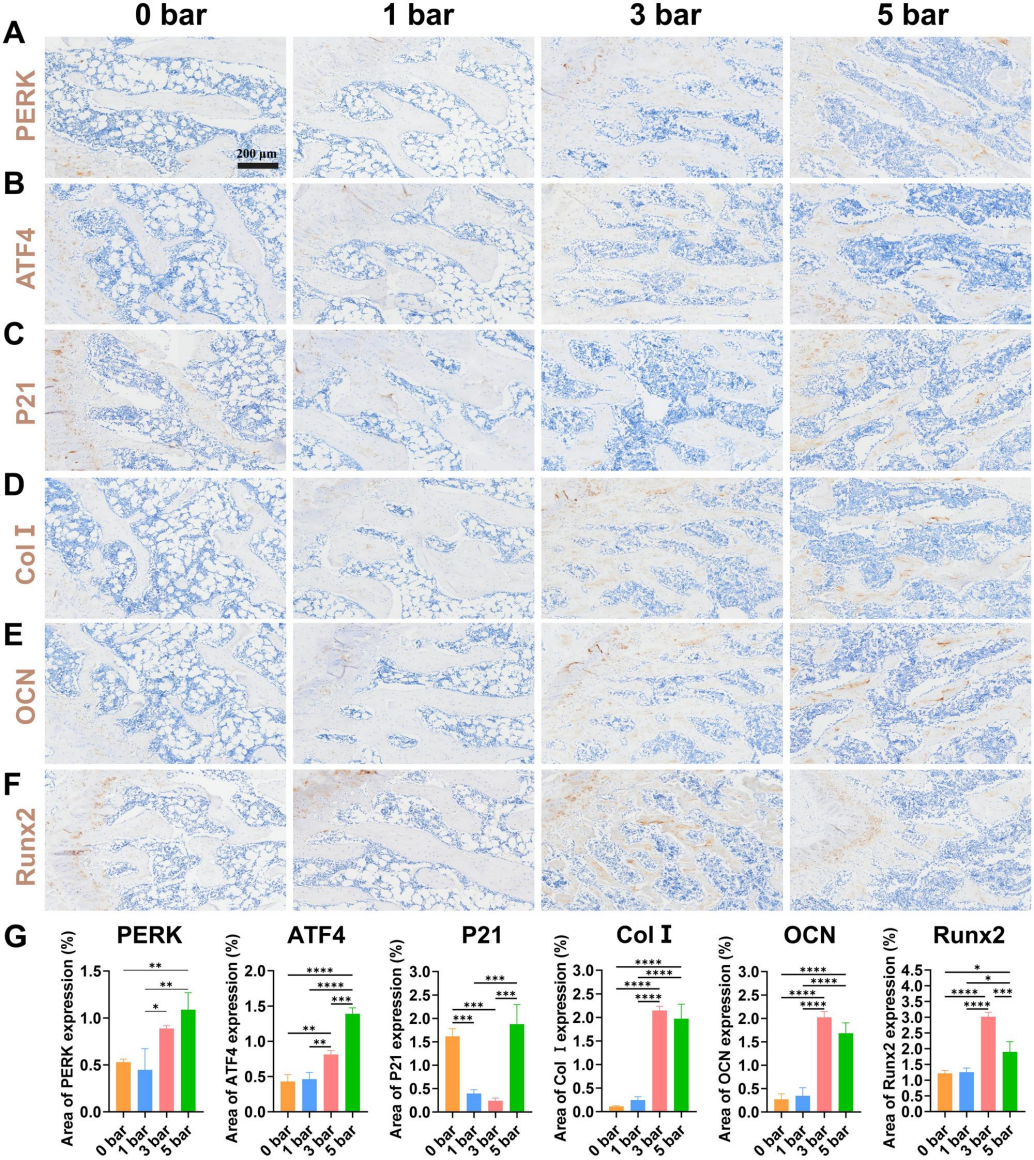
Endoplasmic reticulum stress, inflammation and osteogenesis *in vivo* after treating with different r-ESW intensities. (**A**–**F**) Expression of ER stress (PERK and ATF4), senescence (P21) and osteogenesis related proteins (Col I, OCN and Runx2) in osteoporosis after treating with different r-ESW intensities (scale bar = 200 μm). (**G**) Quantitative analysis of immunohistochemistry. The data are presented as the mean ± SD of *n* = 3. The significance of the data was calculated by the one-way analysis of variance (ANOVA). *P ＜ 0.05, **P ＜ 0.01, ***P ＜ 0.001,****P ＜ 0.0001.

Immunohistochemical staining further confirmed the positive effects of r-ESW treatment on osteogenic potential *in vivo*. Specifically, the 3 bar group exhibited a modest increase in the number of PERK+ and ATF4+ cells compared to both the control and 1 bar groups. Correspondingly, the 3 bar group exhibited notably higher expression levels of essential osteogenic markers, such as Runx2 and OCN, when compared to both the control and 1 bar groups. In contrast, while the 5 bar group displayed a marked increase in the positive staining area for PERK+ and ATF4+ cells, the expression levels of osteogenic markers were similar to or even lower than, those observed in the 3 bar group. Interestingly, the 5 bar group exhibited a reduced expression of Runx2 compared to the 3 bar group. These results imply that moderate ER stress induced by the 3 bar treatment enhances osteogenic differentiation. However, excessive ER stress observed with the 5 bar treatment appears to inhibit this process. Moreover, analysis of the distal femoral metaphysis revealed that the 3 bar group exhibited significantly fewer p21+ cells compared to the other groups, suggesting the presence of a more favorable microenvironment that reduces cellular senescence. These findings are consistent with the observed changes in gene and protein expression, supporting the hypothesis that moderate r-ESW intensities promote osteogenesis through the regulation of ER stress, while higher intensities may disrupt cellular function and impair osteogenic potential (Figure 9).

The ER stress response driven by the PERK-eIF2α-ATF4 pathway is pivotal in modulating BMP2 expression and fostering bone formations that applying 3 bar intensity r-ESW effectively induces a moderate ER stress response, which activates ATF4 transcription and subsequently regulates downstream osteogenic factors like OCN and related translation targets. Additionally, we hypothesize that a mild ER stress response, coupled with dynamic calcium ion fluctuations, can improve the osteoporotic microenvironment. The observed reduction in P21 levels under 3 bar treatment, as well as the decreased IL-1β expression in the SASP also indicate a positive effect on reducing cell senescence, but the mechanism still needs further investigation. In contrast, exposure to a higher 5 bar intensity induces severe, sustained ER stress, which hinders osteoblasts from restoring ER homeostasis and ultimately led to bone loss.

## Conclusion

This study underscores the therapeutic potential of moderate-intensity r-ESW (3 bar) in promoting osteogenesis under osteoporotic conditions. The 3 bar treatment triggered mild ER stress, which activated the PERK-eIF2α-ATF4 signaling pathway, thereby enhancing osteoblast differentiation, gene expression and bone formation. Additionally, moderate shockwave intensity was shown to mitigate cellular senescence. In contrast, higher intensities (5 bar) induced excessive ER stress, resulting in cellular damage and suppression of osteogenesis. *In vivo*, the 3 bar r-ESW treatment improved bone mass and reduced osteoclast activity in osteoporotic rats. These findings suggest that moderate r-ESW intensities could serve as a promising therapeutic approach for treating osteoporosis and promoting bone regeneration.

## Materials and methods

### Primary osteoblastic cell culture

The study adhered to the guidelines authorized by Sichuan University's Institutional Animal Care and Use Committee (IACUC). Eighteen 3-month-old female SD rats (250.2 ± 10.5 g) were obtained from Chengdu DOSSY Experimental Animals Co., Ltd. After a 14-day acclimatization, rats underwent ovariectomy and were housed for 3 months to develop osteoporotic conditions. Twelve rats were used for experimental procedures, and six for isolating primary osteoblastic cell cultures. Osteoblasts derived from OPOB were isolated following a method adapted from Zhao *et al*. [50]. Three months post-ovariectomy, rats were euthanized and their tibiae were collected. The bones were cleaned, cut into 1–2 mm fragments and washed five times with an antibiotic-antimycotic solution. These fragments were cultured in DMEM (DMEM, Gibco, USA) with 10% FBS (FBS, Gibco, USA) at 37°C and 5% CO_2_, with medium replaced every 3 days. Osteoblasts migrated out of the fragments and attached to the plates. All assays were performed in triplicate.

### r-ESW treatment

A shock wave treatment machine (HMCJ200M-r-ESW, Haomeng, Wuhan, China) was utilized for r-ESW therapy. OPOBs were digested with trypsin, resuspended with 3 × 10^6^ cells in 20 mL conditioned medium and treated with shock waves in a floating culture system with medical ultrasound coupling agents to reduce energy attenuation. Different shock wave intensities (1, 3 and 5 bar) were applied at 5 Hz with 500 impulses, while the control group received 0 bar pressure. The experimental groups differed only in shockwave intensity, while all other conditions were kept constant. The pressure levels for radial ESW were selected based on previous studies [10, 25]. Each treatment lasted 3 min. The optimal energy setting was determined by evaluating its impact on cell proliferation, cytocompatibility and osteogenic potential.

### Biocompatibility evaluation

Cell proliferation was assessed using the CCK-8 Kit (Beyotime Bio, China). OPOBs were seeded at 4 × 10^3^ cells per well in 96-well plates. After 1, 3 and 5 days of incubation with three shock wave intensities, cells were treated with 100 μL medium and 10 μL CCK-8 solution and absorbance was recorded at 450 nm using a microplate reader (Bio-Tek, USA). Additionally, OPOBs were treated with r-ESW in 24-well plates at 1 × 10^4^ cells per well. On Day 1, 3 and 5, cells were stained with Calcein AM/PI buffer (Beyotime Bio, China) and imaged using an IX83 Inverted Microscope (Olympus, Japan).

### Quantitative RT-PCR analyses

On Day 3 and 7, samples were collected to analyze the expression of osteoblast-associated genes (ALP, Runx2, Col I, OCN and OPN) and genes related to the ER stress pathway (PERK, eIF2α, ATF4, GRP78) by RT-qPCR. β-actin was used for normalization. Additionally, the expression of senescence-related genes (P16, P21) and inflammatory cytokines (IL-1β, IL-1α) was assessed. Total RNA was isolated and mRNA was reverse-transcribed using the Hifair^®^ III cDNA Synthesis SuperMix (Yeasen, China). Quantitative PCR was performed on a QuantStudio™ 6 Flex Real-Time PCR System (Thermo Fisher Scientific, USA). Data were analyzed using the ΔΔCT-value method. Primer sequences are listed in Supplementary Table S1.

### Western blot analysis

Total protein was isolated using RIPA lysis buffer with PMSF (Solarbio, China) and protein concentration was measured with a BCA Protein Assay Kit (Solarbio, China). For electrophoresis, 20 μg of protein was loaded onto a gel, separated and transferred to PVDF membranes. Membranes were blocked with sealing solution (Qichun, China), incubated with primary antibodies against β-actin, Col I, OPN, OCN, Runx2, ATF4 and PERK (Proteintech, China), and then, with HRP-conjugated secondary antibodies (Oriscience Bio, China). Protein bands were visualized with the Western Lightning chemiluminescence kit (SuperKine, China) and imaged using a ChemiDoc™ XRS+ system (Bio-Rad, USA). Densitometry was performed with Image Lab software. The relative expression levels of the target proteins were quantified by normalizing their band intensities to the corresponding β-actin levels.

### Immunofluorescence staining

Groups were formed based on intervention (control and r-ESW at 1, 3 and 5 bar) with 2 × 10^4^ OPOBs per group (*N* = 4). After 72 hr, cells were fixed with 4% paraformaldehyde, permeabilized with 0.2% Triton X-100 and blocked with 1% BSA-PBS. Samples were incubated overnight with primary antibodies against Col I, OPN, OCN and Runx2 (Proteintech, China). After treatment with secondary antibodies, nuclei were stained with DAPI. Fluorescence images were captured with an IX83 Inverted Microscope (Olympus, Japan), and fluorescence intensity was measured using ImageJ software.

### ALP and Alizarin Red staining

OPOBs were plated at 5 × 10^4^ cells per well in 24-well plates. After 14 and 21 days of osteogenic induction, osteogenic potential and mineralization were assessed using the BCIP/NBT Alkaline Phosphatase Kit (Beyotime, China) and Alizarin Red solution (Solarbio, China). Alkaline phosphatase staining followed the manufacturer's instructions, and for Alizarin Red staining, cells were fixed with 95% ethanol, incubated with Alizarin Red solution and rinsed. Mineralized calcium nodules were imaged with an IX83 Inverted Microscope (Olympus, Japan), and grayscale values were analyzed using ImageJ software.

### SA-β-gal staining

SA-β-gal staining was used to identify senescent OPOBs following the kit instructions (Beyotime, China). OPOBs were fixed for 20 min, washed with PBS and incubated overnight at 37°C in a CO_2_-free incubator with the staining solution. Senescent cells, positive for SA-β-Gal, exhibited enlarged morphology and a bluish color.

### Flow cytometry apoptosis detection

The cell culture medium was transferred to a centrifuge tube, and OPOBs were rinsed with PBS and digested with trypsin. Apoptosis assays were performed using the Annexin-V-FITC Cell Apoptosis Detection Kit (Beyotime, China). Cells (1 × 10^5^ per sample) were suspended in 195 μL Annexin-V-FITC binding buffer, followed by the addition of 5 μL Annexin V and 10 μL PI. After incubation, PBS was added and the samples were analyzed on a Cytoflex flow cytometer (Beckman, USA).

### Fluorescent dyes loading of ER-Ca^2+^ contact sites

OPOBs were cultured in 24-well plates and treated with r-ESW at varying intensities. To visualize ER-Ca^2+^ contact sites, cells were stained with 1 μmol L^−1^ ER-Tracker™ Green (Thermo Scientific, USA) and 5 μM Rhod-5N, AM (AAT Bioquest, USA), with 0.04% Pluronic^®^ F-127, for 30 min at 37°C. After incubation, the staining solution was replaced with HBSS, and cells were imaged using an IX83 inverted microscope (Olympus, Japan).

### RNA-Seq and data analysis

Total RNA was isolated from OPOB cells treated with r-ESW at 5 Hz and 3 bar intensity using the MolPure^®^ RNA Kit (Yeasen, China). The control group consisted of untreated OPOBs. Sequencing libraries were prepared with the NEBNext^®^ UltraTM RNA Library Prep Kit (NEB, USA), and clustering was performed on a cBot Cluster Generation System (Illumina). Raw data were processed with custom Perl scripts, and clean reads were mapped to the reference genome using Hisat2. featureCounts was used to quantify gene reads, and differential expression analysis was performed using DESeq2. Genes with an adjusted *P* values < 0.05 were considered differentially expressed. GO enrichment and KEGG pathway analysis were conducted with the clusterProfiler R package.

### Animal study

The animal study was authorized by the local Animal Care and Use Committee at the Animal Centre, Sichuan University, Chengdu, China (No.20240821003). A total of 12 adult female Sprague-Dawley (SD) rats, each subjected to ovariectomy to induce osteoporosis for three months. r-ESW therapy was performed on rats under anesthesia. Their fur was shaved on both sides, and coupling gel was applied to minimize energy loss. Over four weeks, the experimental groups received weekly r-ESW therapy at 1, 3 or 5 bar intensity, with 2000 pulses, 5 Hz frequency and 10 mm focal length, targeting the bilateral femoral condyles. Rats not receiving therapy were in the control group.

### Micro-CT analysis

At the conclusion of the experimental period, all animals were euthanized by exsanguination under anesthesia. The distal left femur regions were analyzed using a Quantum GX microCT (PerkinElmer, USA) with a 90 µm voxel size, 80 kV and 3-min scan time. A 1.35 mm thick region, starting 0.45 mm below the growth plate, was selected for analysis and 3D reconstruction. Bone parameters, including bone mineral density (BMD), trabecular bone volume per tissue volume (BV/TV), bone surface to bone volume ratio (BS/BV), trabecular number (Tb.N), trabecular separation (Tb.Sp) and trabecular thickness (Tb.Th) were calculated using CTAn software (Brucker) and additional 3D reconstruction was performed with CTvox software.

### Histological and immunohistochemistry analysis

Bone specimens were fixed in 4% paraformaldehyde for 48 hr, decalcified in 10% EDTA for four weeks, and then, dehydrated, paraffin-embedded and sectioned into 5 µm slices. The sections were stained with Methylene Blue/Acid Fuchsin and Masson’s trichrome. For immunohistochemistry, dewaxed sections were treated with 1% citrate antigen retrieval solution at 95°C for 10 min, blocked with 3% hydrogen peroxide and incubated with blocking solution. Primary antibodies against Col I, Runx2, OCN, ATF4, PERK and P21 (1:200) were applied overnight. After rinsing, secondary antibodies and HRP-labeled streptavidin were applied. Color development was performed with DAB-HRP, and nuclei were stained with hematoxylin. Sections were mounted, and positive staining was identified by brown color. Images were captured using a SLIDEVIEW VS200 scanner (Olympus, Japan) at 20× magnification.

### TRAP staining

TRAP activity was assessed using a TRAP staining kit (Jiancheng Co., Ltd., Nanjing, China). For histomorphometric analysis, four random fields of view were selected from a 1000 µm segment above the growth plate in the distal femoral metaphysis, imaged using a SLIDEVIEW VS200 scanner (Olympus, Japan) at 20× magnification.

### Statistics

Statistical analysis was performed using GraphPad Prism 9.5 (GraphPad Software, CA, USA). Data are expressed as the mean ± standard deviation (SD). To compare multiple groups, one-way analysis of variance (ANOVA) followed by Tukey’s test, or two-way ANOVA with Bonferroni’s *post hoc* test, was used. A *P* value of less than 0.05 was considered statistically significant.

## Supplementary Material

rbaf069_Supplementary_Data

## References

[1] Fan Y , Li Q, Liu Y, Miao J, Zhao T, Cai J, Liu M, Cao J, Xu H, Wei L, Li M, Shen C. Sex- and age-specific prevalence of osteopenia and osteoporosis: sampling survey. JMIR Public Health Surveill 2024;10:e48947. 38578689 10.2196/48947 PMC11031699

[2] Lan Z , Lin X, Xue D, Yang Y, Saad M, Jin Q. Can bisphosphonate therapy reduce overall mortality in patients with osteoporosis? A meta-analysis of randomized controlled trials. Clin Orthop Relat Res 2025;483:91–101. 39172899 10.1097/CORR.0000000000003204 PMC11658732

[3] Geusens P , Bours SPG, Wyers CE, van den Bergh JP. Fracture liaison programs. Best Pract Res Clin Rheumatol 2019;33:278–89. 31547983 10.1016/j.berh.2019.03.016

[4] Osnes EK , Lofthus CM, Meyer HE, Falch JA, Nordsletten L, Cappelen I, Kristiansen IS. Consequences of hip fracture on activities of daily life and residential needs. Osteoporos Int 2004;15:567–74. 14730422 10.1007/s00198-003-1583-0

[5] Li N , Cornelissen D, Silverman S, Pinto D, Si L, Kremer I, Bours S, de Bot R, Boonen A, Evers S, van den Bergh J, Reginster JY, Hiligsmann M. An updated systematic review of cost-effectiveness analyses of drugs for osteoporosis. Pharmacoeconomics 2021;39:181–209. 33026634 10.1007/s40273-020-00965-9 PMC7867562

[6] Khosla S , Hofbauer LC. Osteoporosis treatment: recent developments and ongoing challenges. Lancet Diabetes Endocrinol 2017;5:898–907. 28689769 10.1016/S2213-8587(17)30188-2 PMC5798872

[7] Alvarez L. Extracorporeal shockwave therapy for musculoskeletal pathologies. Vet Clin North Am Small Anim Pract 2022;52:1033–42. 35715112 10.1016/j.cvsm.2022.03.007

[8] Kuo SJ , Su IC, Wang CJ, Ko JY. Extracorporeal shockwave therapy (ESWT) in the treatment of atrophic non-unions of femoral shaft fractures. Int J Surg 2015;24:131–4. 26166737 10.1016/j.ijsu.2015.06.075

[9] Chen J , Zhao Q, Zhang X, Meng Q, Shu J, Shao L, Ye G, Guo W. Recent advances in smart biomaterials based on ultrasonic effects. Chem Eng J 2025;507:160524.

[10] Inoue S , Hatakeyama J, Aoki H, Kuroki H, Niikura T, Oe K, Fukui T, Kuroda R, Akisue T, Moriyama H. Effects of ultrasound, radial extracorporeal shock waves, and electrical stimulation on rat bone defect healing. Ann N Y Acad Sci 2021;1497:3–14. 33619772 10.1111/nyas.14581

[11] van der Jagt OP , Piscaer TM, Schaden W, Li J, Kops N, Jahr H, van der Linden JC, Waarsing JH, Verhaar JA, de Jong M, Weinans H. Unfocused extracorporeal shock waves induce anabolic effects in rat bone. J Bone Joint Surg Am 2011;93:38–48. 21209267 10.2106/JBJS.I.01535

[12] Gerdesmeyer L , Schaden W, Besch L, Stukenberg M, Doerner L, Muehlhofer H, Toepfer A. Osteogenetic effect of extracorporeal shock waves in human. Int J Surg 2015;24:115–9. 26455534 10.1016/j.ijsu.2015.09.068

[13] Huang HM , Li XL, Tu SQ, Chen XF, Lu CC, Jiang LH. Effects of roughly focused extracorporeal shock waves therapy on the expressions of bone morphogenetic protein-2 and osteoprotegerin in osteoporotic fracture in rats. Chin Med J (Engl) 2016;129:2567–75. 27779163 10.4103/0366-6999.192776 PMC5125335

[14] Liang W , Chen K, Lv L, Wang Y, Kong J, Liang H, Zhang H, Zhang J, Chen Z, Chang Y-n, Li J, Xing G, Xing G. Radial extracorporeal shock wave responsive precise nanoplatform for effective osteoporosis sequential treatment. Chem Eng J 2021;425:130687.

[15] Hu Y , Tian H, Chen W, Liu Y, Cao Y, Pei H, Ming C, Shan C, Chen X, Dai Z, Yang S, Shao Z, Lan S, Liu Y, Tong W. The critical role of the Piezo1/β-catenin/ATF4 axis on the stemness of Gli1(+) BMSCs during simulated microgravity-induced bone loss. Adv Sci (Weinh) 2023;10:e2303375. 37759400 10.1002/advs.202303375 PMC10646271

[16] Li J , Yang S, Li X, Liu D, Wang Z, Guo J, Tan N, Gao Z, Zhao X, Zhang J, Gou F, Yokota H, Zhang P. Role of endoplasmic reticulum stress in disuse osteoporosis. Bone 2017;97:2–14. 27989543 10.1016/j.bone.2016.12.009

[17] Chueh KS , Juan TJ, Lu JH, Wu BN, Lin RJ, Mao JW, Lin HY, Chuang SM, Chang CY, Shen MC, Sun TW, Juan YS. Low-intensity extracorporeal shock wave therapy ameliorates detrusor hyperactivity with impaired contractility via transient potential vanilloid channels: a rat model for ovarian hormone deficiency. Int J Mol Sci 2024;25:4927. 38732143 10.3390/ijms25094927 PMC11084446

[18] Wang B , Ning H, Reed-Maldonado AB, Zhou J, Ruan Y, Zhou T, Wang HS, Oh BS, Banie L, Lin G, Lue TF. Low-Intensity extracorporeal shock wave therapy enhances Brain-Derived neurotrophic factor expression through PERK/ATF4 signaling pathway. Int J Mol Sci 2017;18:433. 28212323 10.3390/ijms18020433 PMC5343967

[19] Wang B , Zhou J, Banie L, Reed-Maldonado AB, Ning H, Lu Z, Ruan Y, Zhou T, Wang HS, Oh BS, Wang G, Qi SL, Lin G, Lue TF. Low-intensity extracorporeal shock wave therapy promotes myogenesis through PERK/ATF4 pathway. Neurourol Urodyn 2018;37:699–707. 28763567 10.1002/nau.23380 PMC5794657

[20] Saito A , Ochiai K, Kondo S, Tsumagari K, Murakami T, Cavener DR, Imaizumi K. Endoplasmic reticulum stress response mediated by the PERK-eIF2(alpha)-ATF4 pathway is involved in osteoblast differentiation induced by BMP2. J Biol Chem 2011;286:4809–18. 21135100 10.1074/jbc.M110.152900 PMC3039352

[21] Franceschi RT , Ge C, Xiao G, Roca H, Jiang D. Transcriptional regulation of osteoblasts. Ann N Y Acad Sci 2007;1116:196–207. 18083928 10.1196/annals.1402.081

[22] Komori T. Regulation of osteoblast differentiation by transcription factors. J Cell Biochem 2006;99:1233–9. 16795049 10.1002/jcb.20958

[23] Yang X , Matsuda K, Bialek P, Jacquot S, Masuoka HC, Schinke T, Li L, Brancorsini S, Sassone-Corsi P, Townes TM, Hanauer A, Karsenty G. ATF4 is a substrate of RSK2 and an essential regulator of osteoblast biology; implication for Coffin-Lowry syndrome. Cell 2004;117:387–98. 15109498 10.1016/s0092-8674(04)00344-7

[24] Murakami T , Saito A, Hino S, Kondo S, Kanemoto S, Chihara K, Sekiya H, Tsumagari K, Ochiai K, Yoshinaga K, Saitoh M, Nishimura R, Yoneda T, Kou I, Furuichi T, Ikegawa S, Ikawa M, Okabe M, Wanaka A, Imaizumi K. Signalling mediated by the endoplasmic reticulum stress transducer OASIS is involved in bone formation. Nat Cell Biol 2009;11:1205–11. 19767743 10.1038/ncb1963

[25] Inoue S , Hatakeyama J, Aoki H, Kuroki H, Niikura T, Oe K, Fukui T, Kuroda R, Akisue T, Moriyama H. Utilization of mechanical stress to treat osteoporosis: the effects of electrical stimulation, radial extracorporeal shock wave, and ultrasound on experimental osteoporosis in ovariectomized rats. Calcif Tissue Int 2021;109:215–29. 33751141 10.1007/s00223-021-00831-6

[26] Li B , Wang R, Huang X, Ou Y, Jia Z, Lin S, Zhang Y, Xia H, Chen B. Extracorporeal shock wave therapy promotes osteogenic differentiation in a rabbit osteoporosis model. Front Endocrinol (Lausanne) 2021;12:627718. 33841330 10.3389/fendo.2021.627718 PMC8027252

[27] Zhao Z , Wang Y, Wang Q, Liang J, Hu W, Zhao S, Li P, Zhu H, Li Z. Radial extracorporeal shockwave promotes subchondral bone stem/progenitor cell self-renewal by activating YAP/TAZ and facilitates cartilage repair in vivo. Stem Cell Res Ther 2021;12:19. 33413606 10.1186/s13287-020-02076-w PMC7792202

[28] Wang B , Shao W, Zhao Y, Li Z, Wang P, Lv X, Chen Y, Chen X, Zhu Y, Ma Y, Han L, Wu W, Feng Y. Radial extracorporeal shockwave promotes osteogenesis-angiogenesis coupling of bone marrow stromal cells from senile osteoporosis via activating the Piezo1/CaMKII/CREB axis. Bone 2024;187:117196. 39004161 10.1016/j.bone.2024.117196

[29] Hochstrasser T , Frank H-G, Schmitz C. Dose-dependent and cell type-specific cell death and proliferation following in vitro exposure to radial extracorporeal shock waves. Sci Rep 2016;6:30637. 27477873 10.1038/srep30637 PMC4967921

[30] Lv F , Li Z, Jing Y, Sun L, Li Z, Duan H. The effects and underlying mechanism of extracorporeal shockwave therapy on fracture healing. Front Endocrinol 2023;14:1188297. 10.3389/fendo.2023.1188297 PMC10246855 37293486

[31] Schleusser S , Song J, Stang FH, Mailaender P, Kraemer R, Kisch T. Blood flow in the scaphoid is improved by focused extracorporeal shock wave therapy. Clin Orthop Relat Res 2020;478:127–35. 31592777 10.1097/CORR.0000000000000993 PMC7000044

[32] Zhu M , Zhou S, Huang Z, Wen J, Li H. Ca2+-dependent endoplasmic reticulum stress regulates mechanical stress-mediated cartilage thinning. J Dent Res 2016;95:889–96. 27053115 10.1177/0022034516640206

[33] Jiang W , Chen H, Lin Y, Cheng K, Zhou D, Chen R, Song C, Zeng L, Yu H. Mechanical stress abnormalities promote chondrocyte senescence—the pathogenesis of knee osteoarthritis. Biomed Pharmacother 2023;167:115552. 37748410 10.1016/j.biopha.2023.115552

[34] Guo C , Ma R, Liu X, Xia Y, Niu P, Ma J, Zhou X, Li Y, Sun Z. Silica nanoparticles induced endothelial apoptosis via endoplasmic reticulum stress-mitochondrial apoptotic signaling pathway. Chemosphere 2018;210:183–92. 29990757 10.1016/j.chemosphere.2018.06.170

[35] Jiang Z , Wang H, Wang X, Duo H, Tao Y, Li J, Li X, Liu J, Ni J, Wu EJ, Xiang H, Guan C, Wang X, Zhang K, Zhang P, Hou Z, Liu Y, Wang Z, Su B, Li B, Hao Y, Li B, Wu X. TMED4 facilitates regulatory T cell suppressive function via ROS homeostasis in tumor and autoimmune mouse models. J Clin Invest 2024;135:e179874. 39480507 10.1172/JCI179874 PMC11684806

[36] Li Z , Huang Z, Zhang H, Lu J, Wei Y, Yang Y, Bai L. IRE1-mTOR-PERK axis coordinates autophagy and ER stress-apoptosis induced by P2X7-mediated Ca^2+^ influx in osteoarthritis. Front Cell Dev Biol 2021;9:695041. 34222263 10.3389/fcell.2021.695041 PMC8248364

[37] Yang H , Wen Y, Zhang M, Liu Q, Zhang H, Zhang J, Lu L, Ye T, Bai X, Xiao G, Wang M. MTORC1 coordinates the autophagy and apoptosis signaling in articular chondrocytes in osteoarthritic temporomandibular joint. Autophagy 2020;16:271–88. 31007149 10.1080/15548627.2019.1606647 PMC6984599

[38] Li L , Wang H, Zhang J, Sha Y, Wu F, Wen S, He L, Sheng L, You Q, Shi M, Liu L, Zhou H. SPHK1 deficiency protects mice from acetaminophen-induced ER stress and mitochondrial permeability transition. Cell Death Differ 2020;27:1924–37. 31827236 10.1038/s41418-019-0471-x PMC7244772

[39] Rozpedek W , Pytel D, Mucha B, Leszczynska H, Diehl JA, Majsterek I. The role of the PERK/eIF2α/ATF4/CHOP signaling pathway in tumor progression during endoplasmic reticulum stress. Curr Mol Med 2016;16:533–44. 27211800 10.2174/1566524016666160523143937 PMC5008685

[40] Bahar E , Kim H, Yoon H. ER Stress-mediated signaling: action potential and Ca(2+) as key players. Int J Mol Sci 2016;17:1558. 27649160 10.3390/ijms17091558 PMC5037829

[41] Zhou ZY , Sun LQ, Han XY, Wang YJ, Xie ZS, Xue ST, Li ZR. Efficacy, mechanism, and structure-activity relationship of 6-methoxy benzofuran derivatives as a useful tool for senile osteoporosis. J Med Chem 2023;66:1742–60. 36662031 10.1021/acs.jmedchem.2c01377

[42] Yu CL , Lee HL, Yang SF, Wang SW, Lin CP, Hsieh YH, Chiou HL. Protodioscin induces mitochondrial apoptosis of human hepatocellular carcinoma cells through eliciting ER Stress-Mediated IP3R targeting Mfn1/Bak expression. J Hepatocell Carcinoma 2022;9:327–41. 35496076 10.2147/JHC.S355027 PMC9049873

[43] Fernández A , Ordóñez R, Reiter RJ, González-Gallego J, Mauriz JL. Melatonin and endoplasmic reticulum stress: relation to autophagy and apoptosis. J Pineal Res 2015;59:292–307. 26201382 10.1111/jpi.12264

[44] Wu X , Wang Y, Fan X, Xu X, Sun W. Extracorporeal shockwave relieves endothelial injury and dysfunction in steroid-induced osteonecrosis of the femoral head via miR-135b targeting FOXO1: in vitro and in vivo studies. Aging (Albany NY) 2022;14:410–29. 34996049 10.18632/aging.203816 PMC8791199

[45] Zhu Y , Li Z, Sun X, Gao Y, Kang K, He J, Wu Y. Magnetic nanoparticle-infiltrated hydroxyapatite scaffolds accelerate osteoclast apoptosis by inhibiting autophagy-aggravated ER stress. J Mater Chem B 2022;10:8244–57. 36131638 10.1039/d2tb01392d

[46] Pignolo RJ , Law SF, Chandra A. Bone aging, cellular senescence, and osteoporosis. JBMR Plus 2021;5:e10488. 33869998 10.1002/jbm4.10488 PMC8046105

[47] Vetrano M , Ranieri D, Nanni M, Pavan A, Malisan F, Vulpiani MC, Visco V. Hyaluronic acid (HA), platelet-rich plasm and extracorporeal shock wave therapy (ESWT) promote human chondrocyte regeneration in vitro and ESWT-mediated increase of CD44 expression enhances their susceptibility to HA treatment. PLoS One 2019;14:e0218740. 31251756 10.1371/journal.pone.0218740 PMC6599220

[48] Xu J , Yu L, Liu F, Wan L, Deng Z. The effect of cytokines on osteoblasts and osteoclasts in bone remodeling in osteoporosis: a review. Front Immunol 2023;14:1222129. 37475866 10.3389/fimmu.2023.1222129 PMC10355373

[49] Shen G , Ren H, Shang Q, Qiu T, Yu X, Zhang Z, Huang J, Zhao W, Zhang Y, Liang D, Jiang X. Autophagy as a target for glucocorticoid-induced osteoporosis therapy. Cell Mol Life Sci 2018;75:2683–93. 29427075 10.1007/s00018-018-2776-1 PMC11105583

[50] Zhao R , Xie P, Zhang K, Tang Z, Chen X, Zhu X, Fan Y, Yang X, Zhang X. Selective effect of hydroxyapatite nanoparticles on osteoporotic and healthy bone formation correlates with intracellular calcium homeostasis regulation. Acta Biomater 2017;59:338–50. 28698163 10.1016/j.actbio.2017.07.009

