# Supplementary material for: Shockwave-driven activation of endoplasmic reticulum stress in osteoblasts to enhance bone formation under osteoporotic conditions
Source: Regen Biomater. 2025 Jun 27;12:rbaf069. doi: 10.1093/rb/rbaf069 (PMC12448281; doi:10.1093/rb/rbaf069)
Supplement: rbaf069_Supplementary_Data [file rbaf069_supplementary_data.docx]

**Supplementary information**

**Shockwave-driven activation of endoplasmic reticulum stress in osteoblasts to enhance bone formation under osteoporotic conditions**

Dun Luo^1,^**^†^**, Qian Chen^1,2,^**^†^**, Zhuojie Xiao^1,^ **^†^**, Cong Feng^1^, Ruitao Hu^3^, Yuyi Wang^1^, Ce Zhu^1^, Xi Yang^1^, Limin Liu^1,*^, Xiangfeng Li^1,*^, Xiangdong Zhu^1^, Yueming Song^1^, Xingdong Zhang^1^

1 National Engineering Research Center for Biomaterials, Department of Orthopedic Surgery and Orthopedic Research Institute, West China Hospital, Sichuan University, Chengdu, 610065, China.

2 Affiliated Hospital of North Sichuan Medical College, Nanchong, 637199, China

3 Pittsburgh Institute, Sichuan University, Chengdu, 610065, China

†First author: Dun Luo, Qian Chen and Zhuojie Xiao contributed equally to this work.

***Corresponding authors.**

E-mail addresses: liulimin_spine@163.com (L. Liu), hkdlixiangfeng@163.com (X. Li).

**Table. S1** Sequences of primers for quantitative reverse-transcription polymerase chain reaction

| Primer | Primer sequences | |
| --- | --- | --- |
| Name | Forward (5’-3’) | Reverse (3’-5’) |
| ALP | ATGGTAACGGGCCTGGCTACA | AGTTCTGCTCATGGACGCCGT |
| COL1 | CCAGCTGACCTTCCTGCGCC | CGGTGTGACTCGTGCAGCCA |
| OCN | CCTGGCAGGTGCAAAGCCCA | GGGGGCTGGGGCTCCAAGT |
| OPN | AAGCCTGACCCATCTCAGAA | ATGGCTTTCATTGGAGTTGC |
| RUNX2 | CCCAACTTCCTGTGCTCC | AGTGAAACTCTTGCCTCGTC |
| PERK | AGTCGGTCTTTCTCAGTGGG | CCATGTCGCAATCTGTCAGG |
| ATF4 | TGGCTATGGATGGGTTGGTC | GCTCATCTGGCATGGTTTCC |
| eIF2α | TAATTGTCGGACCTGTGGCT | GCAGGTTCTTCTCTCCCAG |
| GRP78 | GAACCAACTCACGTCCAACC | CTTTCCCAAATACGCCTCGG |
| P21 | GTGATATGTACCAGCCACAGG | CAGACGTAGTTGCCCTCCAG |
| P16 | CGTACCCCGATACAGGTGATG | ATACCGCCAAATACCGCACGA |
| IL-1β | ACCACGCTCTTCTGTCTACTG | CTTGGTGGTTTGCTACGAC |
| IL-1α | AAGTTTGTCATGAATGATTCCCTC | GTCTCACTACCTGTGATGATGAGT |
| β-actin | CCACTGCCGCATCCTCTT | GCATCGGAACCGCTCATT |

**
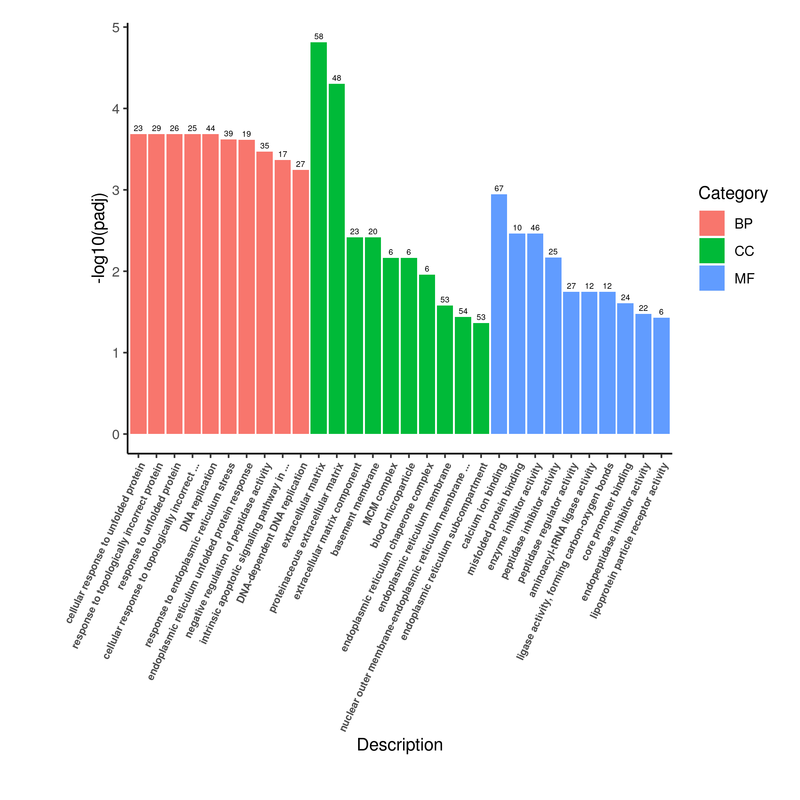
**

**Figure S1.** Bar plot shows the enrichment significance and the number of differentially expressed genes for the 30 most significant GO terms from the GO enrichment analysis.


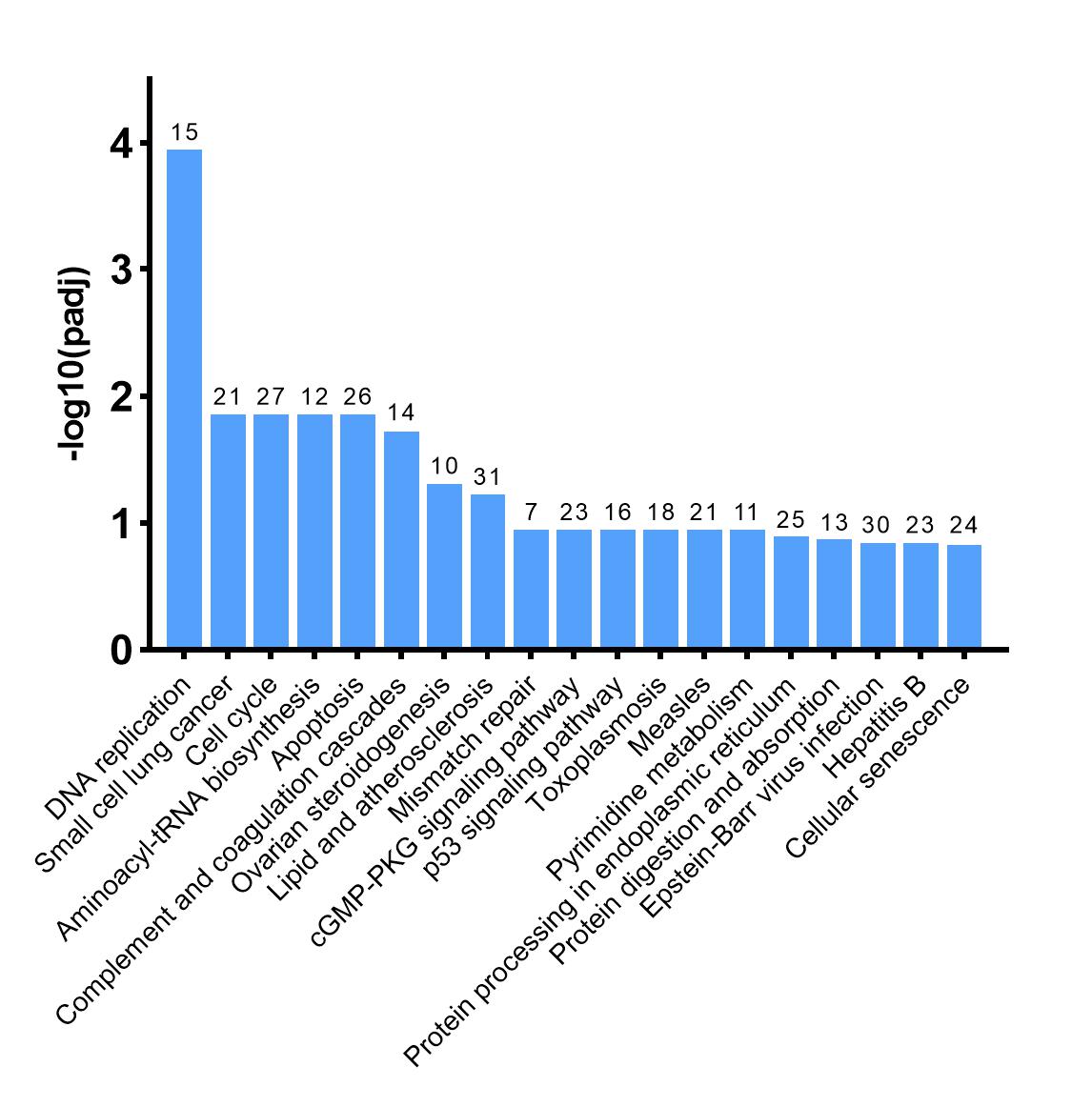


**Figure S2.** Bar plot shows the enrichment significance and the number of differentially expressed genes for the significant pathways from the KEGG enrichment analysis.
